# Impact of ambient temperature on inflammation-induced encephalopathy in endotoxemic mice—role of phosphoinositide 3-kinase gamma

**DOI:** 10.1186/s12974-020-01954-7

**Published:** 2020-10-07

**Authors:** Guang-Ping Lang, Bernadin Ndongson-Dongmo, Trim Lajqi, Michael Brodhun, Yingying Han, Reinhard Wetzker, Martin G. Frasch, Reinhard Bauer

**Affiliations:** 1 Institute of Molecular Cell Biology, Jena University Hospital, Friedrich Schiller University, Hans-Knöll-Straße 2, D-07745 Jena, Germany; 2 Joint International Research Laboratory of Ethnomedicine and Key Laboratory of Basic Pharmacology of Ministry of Education, Zunyi Medical University, Zunyi, 563006 China; 3 Department of Pharmacology, University of Oslo and Oslo University Hospital, Oslo, Norway; 4 Department of Neonatology, University Children’s Hospital, Heidelberg, Germany; 5 Department of Pathology, Helios-Klinikum Erfurt, Erfurt, Germany; 6 Institute of Biochemistry I, Faculty of Medicine, Goethe-University Frankfurt, 60590 Frankfurt, Germany; 7 Department of Anesthesiology and Intensive Care Medicine, Jena University Hospital, Friedrich Schiller University, Jena, Germany; 8 University of Washington School of Medicine, Seattle, WA USA

**Keywords:** Neuroinflammation, Ambient temperature, Microglia, Phosphoinositide 3-kinase γ, Blood–brain barrier, Matrix metalloproteinases, Phagocytosis, Migration

## Abstract

**Background:**

Sepsis-associated encephalopathy (SAE) is an early and frequent event of infection-induced systemic inflammatory response syndrome. Phosphoinositide 3-kinase γ (PI3Kγ) is linked to neuroinflammation and inflammation-related microglial activity. In homeotherms, variations in ambient temperature (T_a_) outside the thermoneutral zone lead to thermoregulatory responses, mainly driven by a gradually increasing sympathetic activity, and may affect disease severity. We hypothesized that thermoregulatory response to hypothermia (reduced T_a_) aggravates SAE in PI3Kγ-dependent manner.

**Methods:**

Experiments were performed in wild-type, PI3Kγ knockout, and PI3Kγ kinase-dead mice, which were kept at neutral (30 ± 0.5 °C) or moderately lowered (26 ± 0.5 °C) T_a_. Mice were exposed to lipopolysaccharide (LPS, 10 μg/g, from *Escherichia coli* serotype 055:B5, single intraperitoneal injection)—evoked systemic inflammatory response (SIR) and monitored 24 h for thermoregulatory response and blood–brain barrier integrity. Primary microglial cells and brain tissue derived from treated mice were analyzed for inflammatory responses and related cell functions. Comparisons between groups were made with one-way or two-way analysis of variance, as appropriate. Post hoc comparisons were made with the Holm–Sidak test or *t* tests with Bonferroni’s correction for adjustments of multiple comparisons. Data not following normal distribution was tested with Kruskal-Wallis test followed by Dunn’s multiple comparisons test.

**Results:**

We show that a moderate reduction of ambient temperature triggers enhanced hypothermia of mice undergoing LPS-induced systemic inflammation by aggravated SAE. PI3Kγ deficiency enhances blood–brain barrier injury and upregulation of matrix metalloproteinases (MMPs) as well as an impaired microglial phagocytic activity.

**Conclusions:**

Thermoregulatory adaptation in response to ambient temperatures below the thermoneutral range exacerbates LPS-induced blood–brain barrier injury and neuroinflammation. PI3Kγ serves a protective role in suppressing release of MMPs, maintaining microglial motility and reinforcing phagocytosis leading to improved brain tissue integrity. Thus, preclinical research targeting severe brain inflammation responses is seriously biased when basic physiological prerequisites of mammal species such as preferred ambient temperature are ignored.

## Background

Sepsis-associated encephalopathy (SAE) is the most common form of encephalopathy occurring in critical care settings and refers to acute neurological dysfunction that arises in the context of extracranial sepsis. SAE is an early feature of infection in the body, occurs quite often with a prevalence of up to 30% in septicemic patients at admission, and SAE severity is associated with increased mortality of septic patients [1]. Although the symptoms of SAE are well recognized—it can take the form of delirium, coma, seizures, or late cognitive decline—its pathophysiology is incompletely understood [2].

Several mechanisms of SAE have been proposed. The hallmarks are thought to comprise diffuse neuroinflammation likely driven by an initial blood–brain barrier (BBB) leakage leading to microglial activation and altered neurotransmission [3]. Activation of cerebral microvascular endothelial cells as the primary constituent of the BBB is regarded as an early event, induced by interaction with a pathogen product such as lipopolysaccharide (LPS) via pattern recognition receptors and proinflammatory factors. The latter include activated complement components and cytokines and lead to an increased endothelial production of reactive oxygen and nitrogen species, accelerated transendothelial cytokine trafficking, and enhanced endothelial permeability [4]. Microglial proinflammatory response further reinforces BBB breakdown and modifies it via PI3Kγ-depended cAMP control [5].

Microglia are considered to be the prototypic tissue-resident macrophage-like innate immune cells of the central nervous system (CNS) that are endowed with memory-like functions to allow context-dependent responses [6–8]. Microglia represent the resident macrophage-like cell population that controls the patterning and wiring of the brain in early development and contributes to homeostasis throughout life [9–12]. Furthermore, microglia implement innate immunity in the CNS as a first line of defense against invading pathogens by continuous micro-environmental surveillance [11, 13–17]. Alterations in brain homeostasis and by most pathologic events induce activation of microglia [18]. During the activation process, microglial cells display specific adaptive functions, including migration towards injury, phagocytosis, antigen presentation, and synapse remodeling [19–21].

Thermoregulation is a fundamental homeostatic function of all mammals; it includes afferent thermal sensing, central regulation, and an efferent response resulting in a tightly controlled body temperature within a narrow species-specific range [22]. Variations of core body temperature (T_C_) outside this range trigger autonomic thermoregulatory responses, mainly via a gradually increased sympathetic activity to minimize radiant heat loss by skin vasoconstriction and maximize heat production by brown adipose tissue thermogenesis [23]. Clinical data clearly indicate that poor outcome of sepsis is associated with spontaneous T_C_ lowering (hypothermia indicating energy depletion) [24–26]. Subgroups of patients with increased risk to develop sepsis such as trauma or burns fail thermal regulation leading frequently to accidental hypothermia [27, 28]. However, in pathogenesis of SAE, the role of challenged thermoregulation upon exposure to a reduced ambient temperature (T_a_) and the resulting accidental hypothermia have not been studied. Moreover, in mouse, a widely used species for modeling SAE, the temperature range of standard practice in preclinical biomedical research [29] and legal recommendations [30] {NRC, 2011 #4699} can be outside the thermoneutral zone for this species. Consequently, the aim of this study was to examine whether exposure of mice to T_a_ outside their thermoneutral zone (but well within the practiced experimental guidelines) will affect BBB breakdown and brain inflammation triggered by LPS-induced systemic inflammatory response in mice. We hypothesized that a reduced T_a_ exacerbates the inflammation-triggered BBB dysfunction at the system’s, organ, and molecular levels. To examine different traits of PI3Kγ signaling on microglial activation, migration, and phagocytic activity, PI3Kγ-deficient mice [31] and mice carrying a targeted mutation in the PI3Kγ gene causing loss of lipid kinase activity [32] were habituated to neutral or reduced T_a_. Subsequently, these animals were exposed to LPS to induce a systemic inflammatory response syndrome (SIRS). Our results demonstrate that challenging thermoregulation by exposure to reduced T_a_ during SIRS causes enhanced early BBB breakdown. This BBB dysfunction is mediated by PI3Kγ-dependent microglial immune responses during acute systemic inflammation. Thus, preclinical research targeting severe brain inflammation responses is seriously biased when basic physiological prerequisites of mammal species such as preferred ambient temperature are ignored.

## Methods

### Animals and experimental procedures

PI3Kγ knockout mice (PI3Kγ^−/−^) [31] and mice carrying a targeted mutation in the PI3Kγ gene causing loss of lipid kinase activity (PI3Kγ^KD/KD^) [32] were on the C57BL/6 J background for > 10 generations. Age-matched C57BL/6 mice were used as controls. The animals were maintained at 12 h light and dark cycles with free access to food and water. The animal procedures were performed according to the guidelines from Directive 2010/63/EU of the European Parliament on the protection of animals used for scientific purposes. Experiments were approved by the committee of the Thuringian State Government on Animal Research.

In order to ensure appropriate acclimatization, animals were introduced at least 1 week before starting the interventions [33]. Animals were divided into a cohort kept at neutral ambient temperature (29.5 ± 0.5 °C) [34] or another cohort kept at lowered ambient temperature (29.5 ± 0.5 °C) during the whole experimental period for appropriate acclimatization. Therefore, mice cohorts were kept in housing cabinets equipped with humidity control, ambient temperature control, and a reliable day/night cycle to ensure replicable environmental conditions (UniProtect; Fa. Ehret, Emmendingen, Germany). Then, mice received LPS (10 mg/kg, intraperitoneal, from *Escherichia coli* serotype 055:B5, Sigma–Aldrich, St. Louis, USA, Lot #032M4082V) as a single intraperitoneal injection. Additionally, 500 μl saline was injected subcutaneously immediately after LPS administration as well as after 24 h in order to support appropriate fluid resuscitation. Clinical status was assessed at baseline and 24 h after LPS administration according to [35].

As an in vitro correlate of hypothermia and neuroinflammation, primary microglia obtained from wild type, PI3Kγ^−/−^, and PI3Kγ^KD/KD^ were exposed to an incubation temperature (T_Inc_) of 33 °C and LPS (100 ng/ml). (For overview, please refer to Table 1.)

**Table 1:**
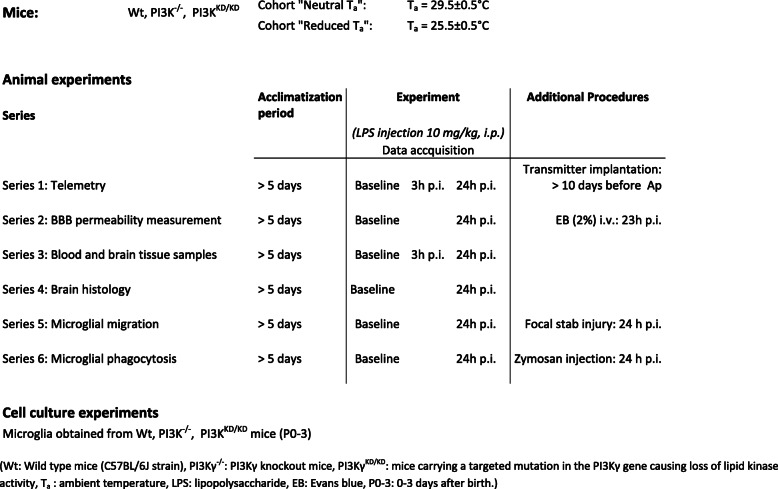
Overview of time course and experimental series

### Telemetric assessment of body core temperature (T_C_) and heart rate

T_C_ and heart rate were assessed by telemetric monitoring of electrocardiography (ECG) and abdominal temperature.

#### Surgical procedure

Mice were anesthetized with 2.5% isoflurane in oxygen. A midline incision was made on the abdomen, and the intraperitoneal cavity was gently opened. An implantable 1.6-g wireless radiofrequency transmitter (ETA-F10, Data Sciences International, St. Paul, MN) was inserted; the leads were transferred through the abdominal wall, and the incision was closed by a surgical suture. The cathodal lead was looped forward subcutaneously to an area overlying the scapula and anchored in place with a permanent suture. The anodal lead was brought subcutaneously to chest near the heart apex. Thereafter, skin incision was sutured. A warming light was used to maintain body temperature between 36 and 37 °C. Meloxicam was given for pain on the day of surgery and the following day. Experiments were initiated 10 days after recovery from surgical instrumentation. Animals were monitored continuously by telemetry by ECG as well as body temperature and motor activity recording.

#### Data acquisition and processing

For simultaneous ECG and body temperature, analog signals were digitalized by the telemetric receiver (model RPC-1, Data Sciences International, St. Paul, MN) and transferred via DSI Data Exchange Matrix at a sampling rate of 2 kHz with 12-bit precision (acquisition software: Ponemah Software 5.20) without a signal filter, and stored on PC for offline data analysis. Instantaneous heart rate (HR) was derived from the reciprocal RR interval time series. Therefore, the individual R-waves, with the R-wave peak as the trigger point, were sequentially recognized (ATISApro®, GJB Datentechnik GmbH, Langewiesen, Germany). Accurate R-wave peak detection was verified by visual inspection. Temperature was continuously measured by the implanted transmitter and stored in parallel to the ECG signal.

### Measurement of blood–brain barrier permeability

BBB disruption was analyzed by measurement of Evans blue (EB) extravasation into brain tissue as described previously [5, 36]. In brief, EB (4 ml/kg of a 2% solution in PBS) was injected through the tail vein 1 h prior to killing. Deeply anesthetized animals were transcardially perfused with ice-cold PBS (40 ml) 24 h after LPS administration. The brains were removed after blood removal, snap-frozen in liquid nitrogen, and stored at − 80 °C. One hemisphere was homogenized in trichloroacetic acid (50 %) and centrifuged (10,000 rpm, 20 min, 4 °C). Supernatant was diluted in three volumes of ethanol. EB was quantified by fluorescence measurement (Tecan Infinite F200, excitation 620 nm, emission 670 nm) and compared to a standard curve. EB concentrations are presented as microgram of EB per microliter of brain tissue supernatant.

### Blood and brain tissue cytokine assessment

The cytokine levels (TNF-α, IL-6, MCP-1, IL-10) in blood and brain tissues were determined using BD™ CBA Mouse Inflammation Kit (Dickinson and Company, San Jose, USA). Blood was obtained via direct heart puncture, collected in a heparinized syringe and immediately centrifuged at 1500×*g* for 10 min at 4 °C. The plasma supernatant was taken immediately and kept at − 80 °C until measurement. The brain tissue was harvested after rinsing with cold PBS, immediately put in liquid nitrogen, and kept at − 80 °C until processing. The brain tissue was then powdered, ice-cold diluted in PBS, homogenated, and centrifuged at 1000 *g* for 10 min at 4 °C. Supernatant was immediately kept at − 80 °C until measurement.

### Primary microglial cell isolation procedure

Neonatal primary microglial cells were isolated from cerebral cortex of newborn mice (6–12 newborn male and female mouse brains were pooled, respectively) as described previously [8, 37, 38]. Briefly, newborn mice were decapitated, and heads were transferred into Petri dishes filled with ice-cold phosphate-buffered saline (PBS). Using fine scissors, the scalp was opened carefully along midline, and the brain was removed. Then, meninges were removed, and cortices and hippocampi were collected in 15-ml tubes filled with PBS. Collected brains were processed in 2 ml dissociation media containing 200 μl 2.5% trypsin and further supplemented with 20 μl of DNAse I in order to digest DNA released from dead cells. After incubation at 37 °C and 5% CO_2_ for 30 min, the medium was removed, and the brain tissues were suspended in 2 ml of Dulbecco’s Modified Eagle’s medium (DMEM, SIGMA-Aldrich #06429, endotoxin tested) containing 10% heat-inactivated fetal bovine serum (FBS, SIGMA-Aldrich #F7524, endotoxin-free and sterile-filtered), 1% Penicillin/Streptomycin, 1% amphotericin B, supplemented with 30 μl DNAse I. Brain tissues were then homogenized and further transferred to T75 cell culture flasks with additional 8 ml culture medium and incubated in 37 °C and 5% CO_2_ for 7 days, followed by medium change and further incubation for 7 more days.

After 14 days, adherent microglial cells were separated from astrocytes by adding PBS-EDTA solution and careful shaking. After harvesting, microglial cells were seeded (75,000 cells/well) in adherent well plates. Purity of microglial cells was between 94 and 98%, as confirmed by the specific Iba1 staining (Suppl. Fig.1). This cell yield and purity are similar to those obtained in previous studies [23, 25].

### RNA extraction and cDNA synthesis

For quantification of mRNA, cells were seeded into 6-well plates and incubated at 37 °C (5% CO_2_) overnight. Afterwards, cells were disintegrated in Trizol reagent (QIAzol Lysis Reagent (#79306), Qiagen, Hilden, Germany). Total RNA was extracted from Trizol as recommended by the manufacturer. To prevent contamination of mRNA preparation with chromosomal DNA, mRNA samples were treated with DNase. RNA amount and purity were determined by Nano-DropTM 1000 (Peqlab, Erlangen, Germany). For first strand cDNA synthesis, 1 μg total RNA was employed using the RevertAid First Strand cDNA Synthesis kit (#K1612) from Thermo Fisher Scientific (Waltham, MA, USA). Synthesis followed the protocol recommended by the manufacturer.

### Quantitative PCR

Quantitative PCR (qPCR) was performed with Maxima SYBR Green/ROX qPCR Master Mix Kit (Fermentas; St. Leon Rot, Germany) containing Maxima Hot Start Taq DNA polymerase and appropriate primer pairs. The following primer pairs were used: MMP-2 forward: TGGCAGTGCAATACCTGAAC and MMP-2 reverse: CCGTACTTGCCATCCTTCTC; MMP-3 forward: GTACCAACCTATTCCTGGTTGC and MMP-3 reverse: CCAGAGAGTTAGATTTGGTGGG; MMP-9 forward: ACCACTAAAGGTCGCTCGGATGGTT, MMP-9 reverse: AGTACTGCTTGCCCAGGAAGACGAA; MMP-13 forward: GGGCTCTGAATGGTTATGACATTC, MMP-13 reverse: AGCGCTCAGTCTCTTCACCTCTT; and GAPDH forward: CATGGCCTTCCGTGTTTCCTA and GAPDH reverse: CCTGCTTCACCACCTTCTTGAT. Relative mRNA expression was calculated in relation to mRNA levels of the housekeeping gene, GAPDH, according to 2-ΔΔCT method [39].

### In vitro chemotaxis assay

To investigate the influence of lipid kinase-dependent and kinase-independent functions of PI3Kγ on microglial migration, transwell assays were performed. Cells were seeded in 6-well plates. After attachment, cells were starved and incubated with intended substances. Following stimulation, 1 × 10^5^ cells were transferred in 300 μl serum-free medium into the upper chamber of a 12-well chemotaxis insert (ThinCertTM, 8 μm pores; Greiner-Bio-One GmbH, Frickenhausen, Germany). The chamber was placed in 700 μl serum-free medium containing chemoattractant (C5a; 10 ng/ml) and incubated at 37 °C (normal T_Inc_) or at 33 °C (reduced T_Inc_) with 5% CO_2_ for 2 h. Afterwards, cells on the lower side of the insert membrane were fixed with 100% ice-cold methanol and stained with 0.5% crystal violet solution (in 25% methanol) for 10 min. Average count of migrated cells was estimated through consideration of five independent visual fields.

### In vivo microglial migration assay

Experiments were performed on adult (10–14 weeks) wild type, PI3Kγ^−/−^, and PI3Kγ^KD/KD^ mice (7 mice per group) kept during the whole experimental period at neutral T_a_ or reduced T_a_, respectively. To investigate the effect of targeted PI3Kγ mutation on microglial migration at neutral or reduced T_a_, an in vivo wound-healing experiment was performed. Mice were anesthetized by intraperitoneal injection of ketamine (100 mg/kg) and xylazine (16 mg/kg), and positioned in a stereotaxic apparatus (Stoelting, Wood Dale, IL, USA). Mice were then placed on a homeothermic heat blanket to maintain normal body temperature during surgery. The skull was exposed by a skin incision, and small burr holes were drilled through the skull. Using a micromanipulator focal stab, an injury was performed by gentle insertion of stainless steel pin (diameter 0.25 mm) into the parietal cortex at 3 mm below the dura mater [40, 41]. The pin was kept in place for 2 min and then removed. The burr holes were covered with bone wax, and the animals were returned to their cages. Twelve hours later, mice were deeply anesthetized and perfused with 4% paraformaldehyde (PFA) in phosphate buffer by cardiac puncture via the left ventricle. Brains were removed immediately after fixation and post-fixed for 5 h in 4% PFA at 4 °C. After cryoprotection in phosphate-buffered saline (PBS) containing 30% sucrose, brains were frozen in methylbutane at − 30 °C and stored at − 80 °C. Whole brains were cut by horizontal sections at 40 μm on a freezing microtome (Microm International GmbH, Thermo Scientific, Germany). The slices were immunostained with anti-Iba1 antibody to visualize microglia. Sections were photographed with a digital fluorescence camera (Nikon DSQi2) and mounted on the Nikon inverted research microscope Eclipse Ti (Nikon Instruments Europe B.V., Amstelveen, The Netherlands). Quantitative measurements (ImageJ software, National Institutes of Health, Bethesda, MD) blinded to the treatment groups were used to count cell numbers per voxel and expressed in cubic millimeter. At the injured region, three voxels were predefined as follows: voxel 1, a cylinder with a diameter of 400 μm, center lying in the middle of injury, and an altitude of 40 μm; voxel 2, hollow cylinder, subsequently on voxel 1, with an inner diameter of 400 μm, an outer diameter of 800 μm, and an altitude of 40 μm; voxel 3, hollow cylinder, subsequently on voxel 2, with an inner diameter of 800 μm, an outer diameter of 1200 μm, and an altitude of 40 μm. Number of Iba1-positive cells was counted in all three voxels. Migratory index was estimated as the ratio of cell number in voxel 1 divided by the sum of cell number in voxels 1, 2, and 3 as described previously [40, 42].

### In vitro phagocytosis assay

Efficiency of phagocytosis at reduced versus neutral T_a_ was investigated as previously described [40, 42]. Briefly, primary microglia cells obtained from wild type, PI3Kγ^−/−^, and PI3Kγ^KD/KD^ mice were seeded into 12-well plates and incubated at 37 °C (5% CO_2_) for 24 h. After attachment, cells were starved for 24 h in DMEM without FCS. Cell were subsequently stimulated with LPS (100 ng/ml) or left unstimulated. Phagocytosis assay was performed by using fluorescein isothiocyanate (FITC)-labeled Zymosan A (*S. cerevisiae*) BioParticles (9800 U/ml) (#Z2841, Thermo Fisher Scientific, Waltham, USA). Seven microliters of the suspended particles was added to the microglial cells and incubated 1 h at either 37 °C or 33 °C. After incubation, the cells were fixed with 4% PFA, washed three times, and stained with DAPI–solution for 5 min (1:1000 in 1× PBS). Phagocytosed particles and cells of five independent visual fields were counted under a fluorescence microscope (Nikon Eclipse Ti, Nikon Instruments—Japan). The result of the phagocytosis of primary microglia was calculated by determining the phagocytic index (the uptake rate of FITC-Zymosan particles per cell).

### In vivo phagocytosis assay

Experiments were performed on adult (10–14 weeks) wild type, PI3Kγ^−/−^, and PI3Kγ^KD/KD^ mice (7 mice per group) kept during the whole experimental period at neutral T_a_ or reduced T_a_, respectively. To investigate the effect of targeted PI3Kγ mutation on microglial phagocytosis, FITC-labeled Zymosan particles (9800 U/ml) were administered into the brain as described previously [40]. Briefly, mice were anesthetized by intraperitoneal injection of ketamine (100 mg/kg) and xylazine (16 mg/kg), and positioned in a stereotaxic apparatus (Stoelting, Wood Dale, IL, USA). The skull was exposed by a skin incision, and small burr holes were drilled through the skull. Using a micromanipulator, a cannula (diameter 0.24 mm) attached on a Hamilton microsyringe (10 μl) was stereotaxically placed into the parietal cortex on both sides (stereotaxic coordinates were AP, − 2.0 mm; L, ± 0.5 mm; and V, − 2.5 mm, respectively [43]). Subsequently, 4 μl of FITC-labeled Zymosan particles suspended in artificial cerebrospinal fluid were infused within 120 s. The cannula remained in place for 5 min before removal. Twenty-four hours later, mice were deeply anesthetized and perfused with 4% PFA in phosphate buffer by cardiac puncture via the left ventricle. Brains were removed immediately after fixation and post-fixed for 5 h in 4% PFA at 4 °C. After cryoprotection in PBS containing 30% sucrose, brains were frozen in methylbutane at − 30 °C and stored at − 80 °C. Whole brains were cut by coronal sections at 40 μm on a freezing microtome (Microm International GmbH, Thermo Scientific, Germany). The slices were immunostained with anti-Iba1 antibody to visualize microglia. A voxel with an edge length of 400 μm and an altitude of 40 μm were predefined as region of interest. Z-stack imaging was performed with a × 20 objective using a digital fluorescence camera (Nikon DS-Qi2), mounted on the Nikon inverted research microscope Eclipse Ti (Nikon Instruments Europe B.V., Amstelveen, The Netherlands). Quantitative measurements (ImageJ software, National Institutes of Health, Bethesda, MD) blinded to the treatment groups were used to count the percentage number of Iba-1 positive cells per cubic millimeters containing Zymosan particles.

### Histopathology and immunohistochemistry

For determination of microglial activation, PMN homing, MMP-9 expression, and TUNEL positivity, brains were fixated in situ by transcardial perfusion with 4% PFA after rinsing with PBS. Afterwards, they were immediately removed after fixation, post-fixated in 4% PFA at 4 °C for 1 day, embedded in paraffin, and cut into 6-μm-thick sections. After deparaffinization, the sections were heated with citrate buffer (0.01 M, pH 6.0) in the microwave (630 W, 11 min) for antigen removal, and the nonspecific binding sites were blocked with blocking solution (5% NDS, 1% BSA-c, PBST). Then, the slide-mounted tissue sections were incubated with the desired primary antibody in antibody incubation solution (5% NDS, 1% BSA-c, PBST) at 4 °C overnight, followed by an incubation with the associated secondary antibody at 4 °C for 1 h. Negative control sections were incubated with goat serum in the absence of the primary antibody. The following primary antibodies were used: goat polyclonal anti-Iba-1 (1:250) antibody (Abcam, Cambridge, UK) for Iba1 staining, rabbit polyclonal anti-MMP-9 (1:150) antibody (Cell Signaling Technology, Danvers, USA) for MMP-9, and rabbit anti-mouse PMN (Accurate Chemical & Scientific CO, USA) for neutrophil staining. For visualization, the secondary fluorescent goat anti-mouse isotype-specific antibody Alexa Fluor® 488 (Molecular Probes, Inc., Eugene, USA) and donkey anti-goat IgG antibody Alexa Fluor®568 (Thermo Fisher Scientific, Waltham, USA) were used. Method for TUNEL staining was described elsewhere [44]. Briefly, sections were deparaffinized and prepared for TUNEL-staining. Fragmented DNA was detected in situ by the TUNEL method using a commercially available kit according to the manufacturer’s protocol (In Situ Cell Death Detection Kit, POD; Roche, Germany). Deparaffinized sections were pretreated with 20 mg/ml proteinase K and washed in PBS prior to TUNEL staining. TUNEL staining was performed by incubation with fluorescein-conjugated digoxigenin-UTP and terminal deoxynucleotidyltransferase at 37 °C for 1 h. DNA fragmentation was visualized using converter-alkaline phosphatase, NBT/BCIP, and counterstaining with Kernechtrot.

#### Cell counting for assessment of microglial cell activation

Cells were classified as ramified, amoeboid, unipolar, and bipolar. Ramified (normal) microglial cells are defined by thin, slender, radially projecting processes with well-developed ramifications. Amoeboid microglial cells are defined as having large soma, and short, thick, and radially projecting processes. Unipolar and bipolar microglial cells were defined as having one or two thick process with well-developed ramifications [14, 45]. Estimation of cell counting migration and phagocytic index were performed by a co-author (G.-P. L.) blinded for genotype and treatment. In each case, evaluation was performed on three different slices obtained from the frontal cortex, thalamus, and hippocampus, each. Five separate fields of vision were counted with at least 100 cells.

### Statistics

The statistical analysis was performed using the SigmaPlot Software (Sigma-Plot Software, San Jose, USA). All data are presented as boxplots illustrating medians within boxes from first quartile (25th percentile) to the third quartile (75th percentile) and whiskers ranging from the 10th to the 90th percentiles (extreme values are marked outside). Numbers of animals are given in figure legends for each group and time point. Comparisons between groups were made with one-way or two-way analysis of variance, if appropriate. In case of repeated measurements, one-way and two-way analysis of variance with repeated measures was used, if appropriate. Post hoc comparisons were made with the Holm–Sidak test or *t* tests with Bonferroni’s correction for adjustments of multiple comparisons. Data not following normal distribution was tested with Kruskal-Wallis test followed by Dunn’s multiple comparisons test.

## Results

### Impact of ambient temperature on degree of SIRS and SIRS-induced BBB disturbance

**Fig. 1:**
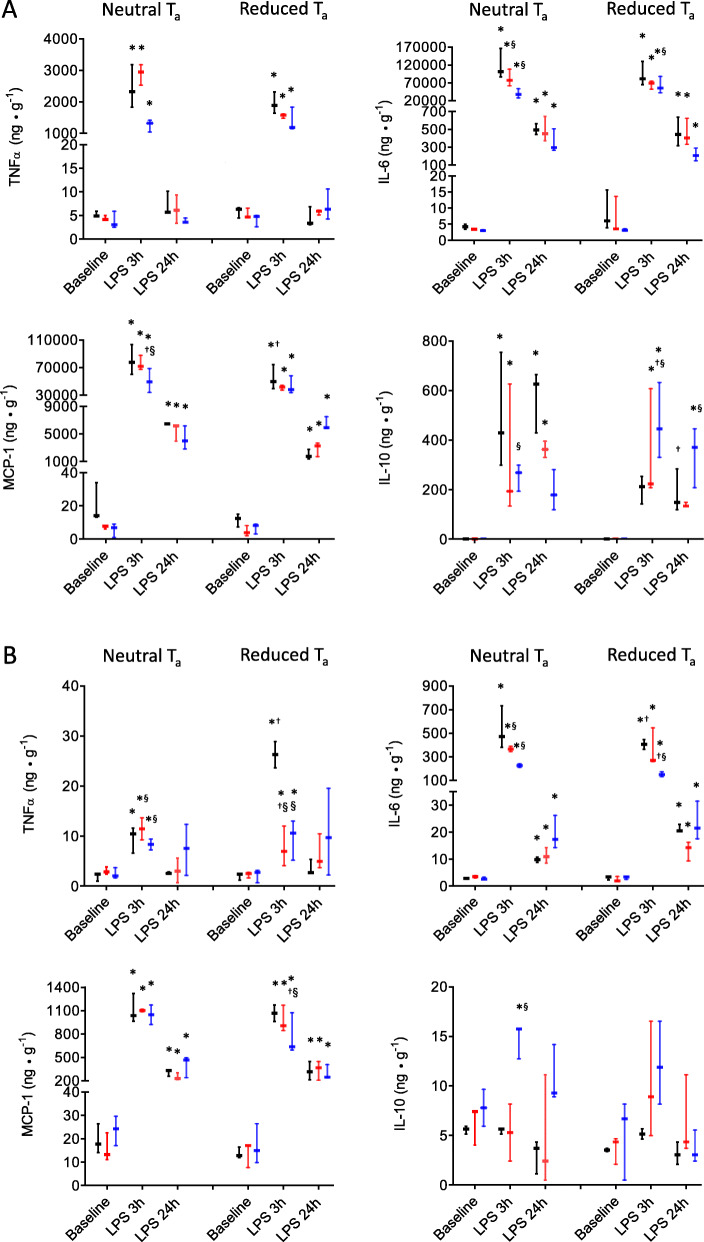
Cytokine levels in blood plasma (**a**) and brain tissue (**b**). Values are given as medians and whiskers (minimum and maximum), *n* = 3, for each group and experimental state. * ^† §^ *p* < 0.05, asterisk indicates significant differences versus baseline conditions within the same genotype, dagger sign significant differences versus neutral Ta within the same genotype, section sign indicates significant differences versus wild-type (Wt) mice within the same Ta condition

Intraperitoneal LPS administration induced a robust SIRS in mice kept under neutral as well as reduced T_a_ as revealed by cytokine release in blood plasma and brain tissue (Fig. 1). IL-10 anti-inflammatory response was barely developed. However, reduced T_a_ induced a worsened sickness state of SIRS in PI3Kγ-deficient mice as measured by the clinical severity score (Tabl.1 Suppl.).

**Fig. 2:**
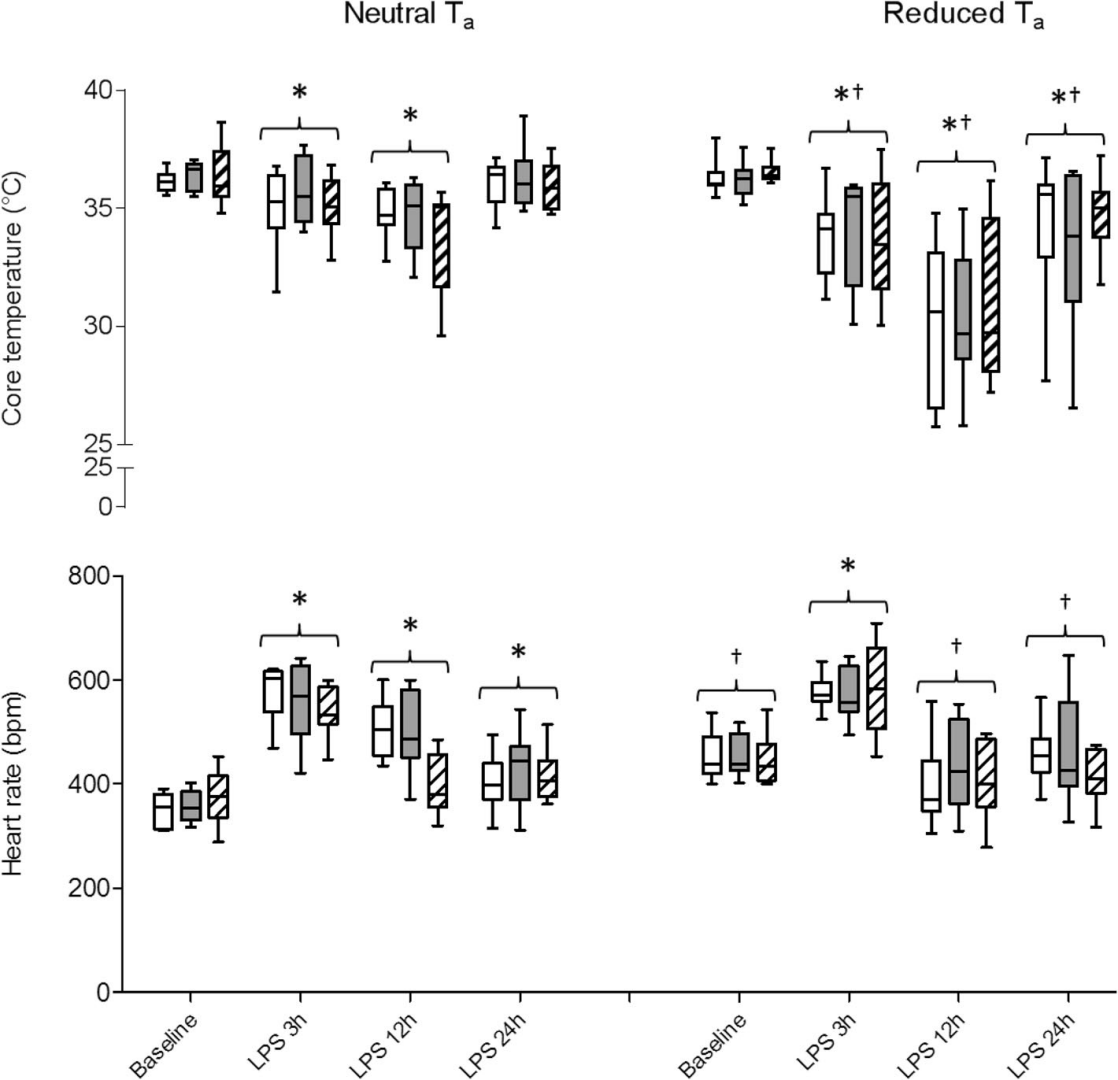
Altered heart rate and augmented hypothermia in mice kept under reduced ambient temperature (T_a_) after LPS-induced SIRS response compared with mice kept under neutral T_a_ irrespective of the genotype (wild-type mice, open boxplots, PI3Kγ-deficient mice (PI3Kγ^−/−^) filled boxplots, PI3Kγ-kinase-dead mice (PI3Kγ^KD/KD^) hatched boxplots). Values are presented as boxplots illustrating medians within boxes from first quartile to the third quartile, whiskers ranging from the 10th to the 90th percentiles (neutral T_a_ groups: wild-type mice *n* = 9, PI3Kγ^−/−^ *n* = 10, PI3Kγ^KD/KD^, *n* = 8; reduced T_a_ groups: wild-type mice *n* = 12, PI3Kγ^−/−^ *n* = 10, PI3Kγ^KD/KD^ *n* = 9). * ^†^ *p* < 0.05, asterisk indicates significant difference between baseline and LPS stimulation within each T_a_ state, dagger sign significant differences versus mice kept under neutral T_a_ (two-way repeated measures ANOVA, followed by Holm–Sidak test for post hoc multiple comparisons were performed)

Telemetric T_C_ monitoring revealed that under baseline conditions, all mice, regardless T_a_, showed no differences in body temperature. However, reduced T_a_ was accompanied by an enhanced sympathetic tone to the heart already under baseline conditions, indicated by an increased HR regardless of the genotype (Fig. 2). Importantly, whereas the early period of LPS-induced SIRS was characterized by a rather similar HR elevation, HR remained elevated in the animals kept under neutral T_a_, but not in the animals kept under reduced T_a_, throughout the observation period. Furthermore, all mice kept under neutral T_a_ exhibited a short-term period of mild hypothermia whereas the mice kept under reduced T_a_ developed a markedly more pronounced and longer (24 h versus 12 h) lasting hypothermic period (Fig. 2).

**Fig. 3:**
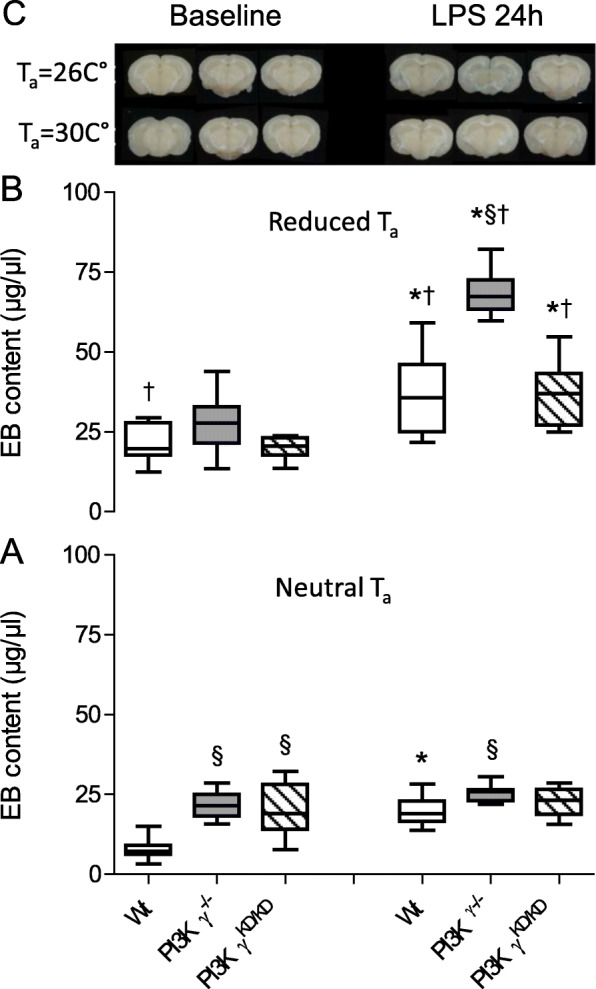
Enhanced BBB leakage in PI3Kγ-deficient mice at reduced T_a_ 24 h after LPS-induced SIRS. **a** Mice kept under neutral T_a_ show mild disturbance of BBB integrity in PI3Kγ mutant. LPS-induced SIRS elicits small increase of BBB leakage in wild-type mice (Wt, open boxplots). **b** Reduced T_a_ was accompanied by enhanced Evans blue (EB) extravasation into brain tissue indicating degree of BBB leakage in PI3Kγ-deficient (PI3Kγ^−/−^) mice (filled columns) in comparison with Wt and kinase-dead (PI3Kγ^KD/KD^, hatched boxplots) mice. **c** Representative pictures of native brain slices immediately after transcardial rinsing with physiological saline, brain removal, and cutting into coronal slices using a brain matrix. Values are presented as boxplots illustrating medians within boxes from first quartile (25th percentile) to the third quartile (75th percentile) and whiskers ranging from minimum to maximum, *n* = 10 per group and time point. * ^† §^ *p* < 0.05, asterisk indicates significant differences versus baseline conditions within the same genotype, dagger sign indicates significant differences versus neutral T_a_ within the same genotype, section sign indicates significant differences versus Wt mice within the same T_a_ condition (two-way ANOVA and one-way ANOVA, followed by Holm–Sidak test for post hoc multiple comparisons was performed for comparison between respected groups, *t* test was used for comparisons between states within same groups with Bonferroni’s correction for adjustments of multiple comparisons)

Under neutral T_a_, LPS-induced SIRS provoked an increase of BBB leakage in wild type mice, whereas PI3Kγ-deficient mice exhibited a significantly enhanced BBB disturbance compared with wild-type mice (Fig. 3). At baseline, this was also true for PI3Kγ-kinase-dead mice.

In contrast, at baseline, housing under reduced T_a_ induced BBB leakage in wild-type mice to a similar degree as in the mutant mice (Fig. 3b). Moreover, already at baseline, in the wild-type mice, the BBB leakage was more pronounced at reduced T_a_ than at neutral T_a_. LPS-induced SIRS provoked a substantially enhanced BBB leakage, which was most pronounced in PI3Kγ-deficient mice. Of note, lipid kinase-dead mutant mice display similar degree of BBB breakdown as the wild-type mice indicating a causal impact of lipid kinase-independent PI3Kγ signaling on the development of BBB disturbance during SIRS.

### Impact of T_a_ and SIRS on degree of microglial activation, MMP expression, apoptosis, and PMN invasion

**Fig. 4:**
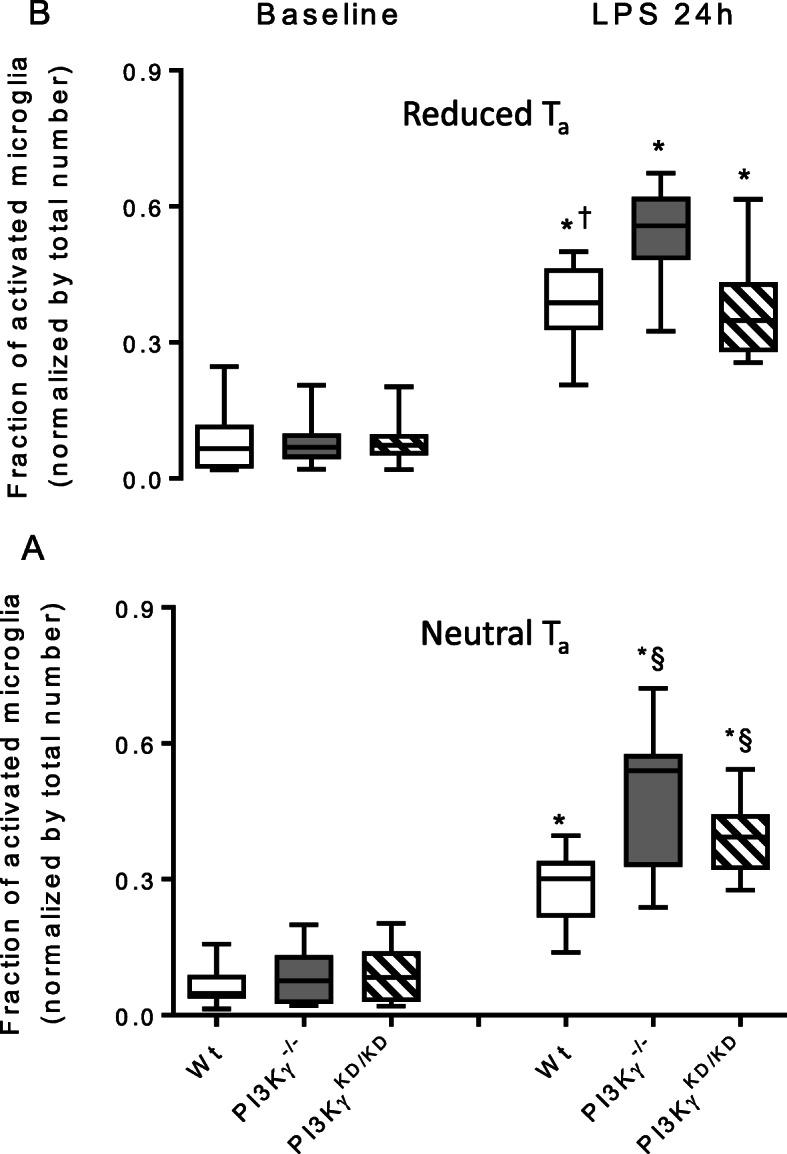
Marked increase of microglial cell activation due to LPS-induced SIRS in brains of mice kept under neutral T_a_ (**a**) and reduced T_a_ (**b**). The effect is exacerbated by reduced T_a_ in the wild-type mice, but not in the genetically modified animals. Values are presented as boxplots illustrating medians within boxes from first quartile to the third quartile and whiskers ranging from the 10th to the 90th percentiles (**a**, **b**: *n* = 4–6, at each group and experimental state. * ^§ †^ *p* < 0.05, asterisk indicates significant differences versus baseline within each group, section sign indicates significant differences versus wild-type mice kept under same T_a_, dagger sign indicates significant differences versus mice kept under neutral T_a_ at the same experimental state, two-way ANOVA, followed by Holm–Sidak test for post hoc multiple comparisons, each)

To verify consequences of T_a_ and LPS-induced SIRS, extent of neuroinflammation was assessed by different approaches. First, we quantified the number of activated microglial cells assessed by shape characteristics [45]. As shown in Fig. 4 and Fig. 2 Suppl., a marked increase in microglial cell number with altered, mainly polarized shape occurred. Analysis of regional distribution revealed similarity in the extent of regional microglial cell activation in the brain cortex, hippocampus, and thalamus (Tabl. 2 Suppl.) suggesting a diffuse microglial activation due to LPS-induced SIRS. While we did not observe a significant genotype-related effect, the wild-type mice showed an exacerbated response with regard to activated microglia counts at reduced T_a_.

**Fig. 5:**
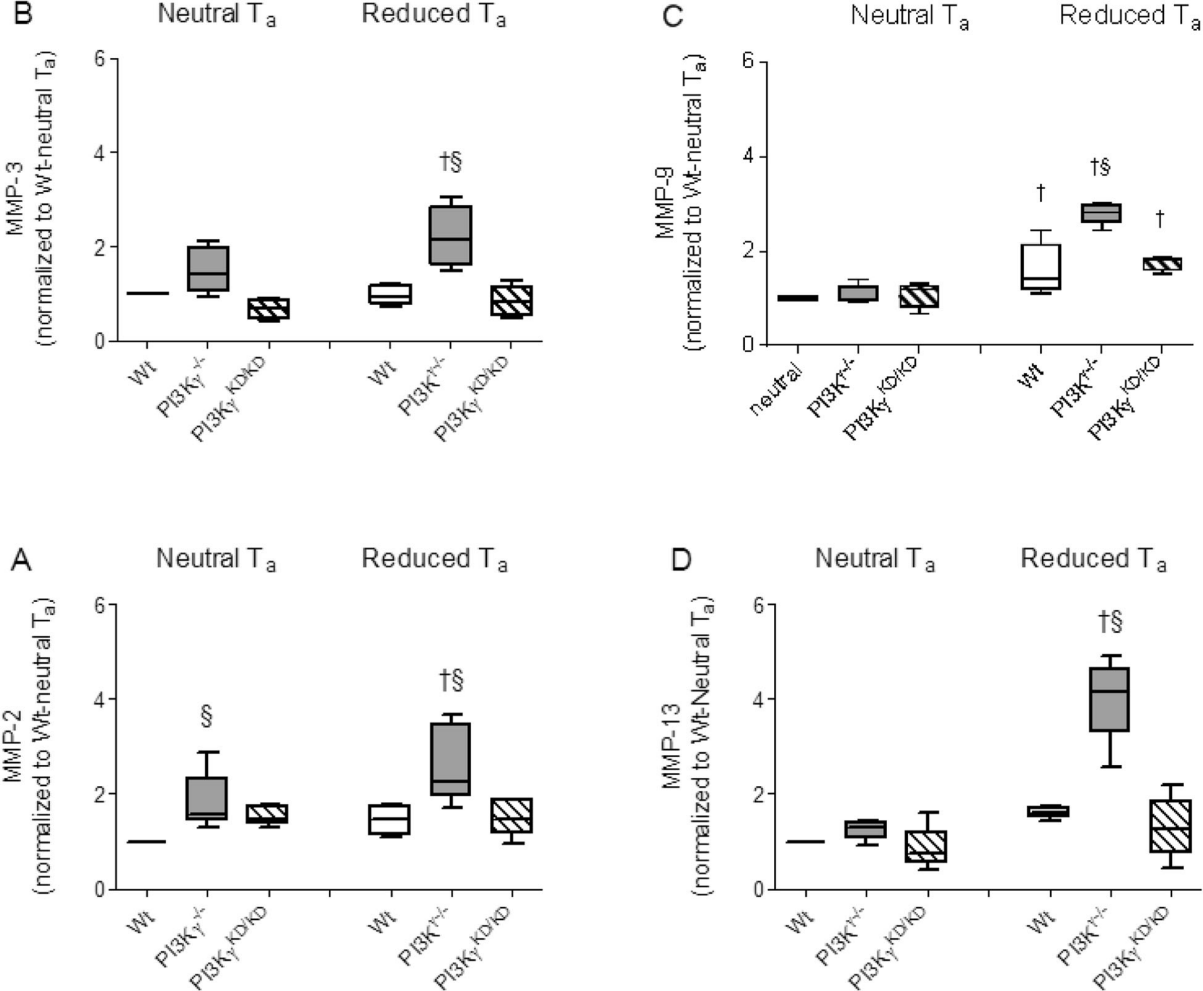
Increased LPS-induced mRNA expression of MMP-2, MMP-3, MMP-9, and MMP-13 appeared 24 h post LPS mainly in brains obtained from PI3Kγ-deficient mice kept under reduced T_a_. Values are presented as boxplots illustrating medians within boxes from first quartile to the third quartile and whiskers ranging from the 10th to the 90th percentiles (**a**–**d**, *n* = 5 per each group and experimental state. ^§ †^ *p* < 0.05, section sign indicates significant differences versus wild-type mice kept under same T_a_, dagger sign indicates significant differences versus mice kept under neutral T_a_ of the same genotype, two-way ANOVA, followed by Holm–Sidak test for post hoc multiple comparisons, each)

In contrast, MMP expression in brain tissue obtained 24 h after LPS injection displayed a significant T_a_ as well as PI3Kγ dependency. There was an enhanced RNA expression in the brains obtained from PI3Kγ-deficient mice kept under reduced T_a_ in all MMPs under consideration compared to mice kept under neutral T_a_ (Fig. 5). Furthermore, there was an increased mRNA expression in brains derived from PI3Kγ-deficient mice kept under reduced T_a_ compared with wild-type mice kept under same housing conditions. In contrast, PI3Kγ^KD/KD^ mice showed a similar response as wild-type mice, again suggesting a lipid kinase-independent mode of action.

**Fig. 6:**
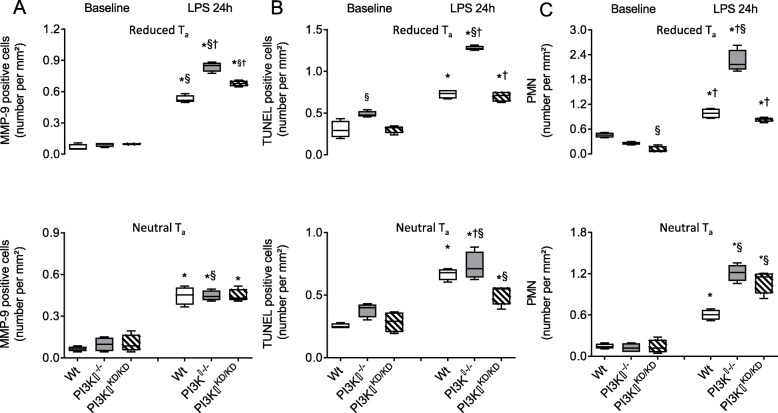
Among the Iba1-positive cells, reduced T_a_ (upper plot) 24 h after LPS administration resulted in an increased number of MMP-9 positive cells (**a**), number of TUNEL positive cells (**b**), and number of invading polymorphonuclear cells (PMN, **c**) appearing mainly in the brains obtained from PI3Kγ-deficient mice. Values are presented as boxplots illustrating medians within boxes from first quartile to the third quartile and whiskers ranging from the 10th to the 90th percentiles (lower panel: neutral T_a_, upper panel: reduced T_a_, *n* = 4 per each group and experimental state. * ^§ †^ *p* < 0.05, asterisk indicates significant differences versus baseline of the same genotype and experimental state, section sign indicates significant differences versus wild type mice kept under same T_a_, dagger sign indicates significant differences versus mice kept under neutral T_a_ at the same experimental state, two-way ANOVA, followed by Holm–Sidak test for post hoc multiple comparisons, each)

Increased cerebral MMP expression appeared as a result of enhanced microglial activation in PI3Kγ^−/−^ mice kept under reduced T_a_ as is evidenced by an increased number of MMP-9 positive cells co-expressed in Iba-1 positive cells in these brains (Fig. 6a). Analysis of regional distribution revealed similarity in the extent of microglial MMP-9 expression in the brain cortex, hippocampus, and thalamus (Tabl. 3 Suppl.), again suggesting a uniform increase in activity.

To examine a possible T_a_-dependent impact on structural integrity of brain tissue due to LPS-induced SIRS, we quantified the extent of apoptosis in brain slices derived from wild type, PI3Kγ^−/−^, and PI3Kγ^KD/KD^ mice kept under neutral and reduced T_a_. Albeit of small magnitude, already under baseline conditions, the rate of apoptotic cells was significantly increased in brains of PI3Kγ^−/−^ mice kept under reduced T_a_ compared with those kept at neutral T_a_. LPS-induced SIRS exhibited consistently an increased number of apoptotic cells, which was most pronounced in PI3Kγ^−/−^ mice kept under reduced T_a_ (Fig. 6b, Fig. 3 Suppl.). Regional comparison revealed that numbers of TUNEL-positive cells in PI3Kγ^−/−^ mice kept under reduced T_a_ were markedly higher in the hippocampus compared to cortex and thalamus (Tabl. 3 Suppl.).

To assess a contribution of blood-born immune cells to pathogenesis of SIRS-induced SAE, we quantified the extent of invading PMN. Whereas under baseline conditions, merely scattered PMN were encountered, and neither T_a_ nor genotype-related effects have been observed; LPS-induced SIRS was accompanied by a distinct increase of invading PMN into the brain tissue. We found a significant T_a_-dependent effect in PI3Kγ-deficient mice observing an enhanced PMN homing into brain tissue in mice kept under reduced T_a_ (Fig. 6c, Fig. 3 Suppl.). However, consistent with findings for Iba1-positive microglia cell numbers and MMP expression, we observed no brain regional differences in PMN invasion (Tabl. 3 Suppl).

### Impact of T_a_ incubation temperature on microglial migration and phagocytosis

The ability to migrate toward different chemotactic stimuli including those released by brain injuries is an important property of microglial cells, which is essential for biological functions. Our previous studies revealed a dependency of lipid kinase-related PI3Kγ signaling on directed motility of microglial cells owing to inflammatory stimulation [40]. Herein, we addressed the question if PI3Kγ-dependent migration of microglia is a result of different ambient temperatures and its in vitro surrogate, i.e., the varied temperatures of incubation (T_Inc_).

**Fig. 7:**
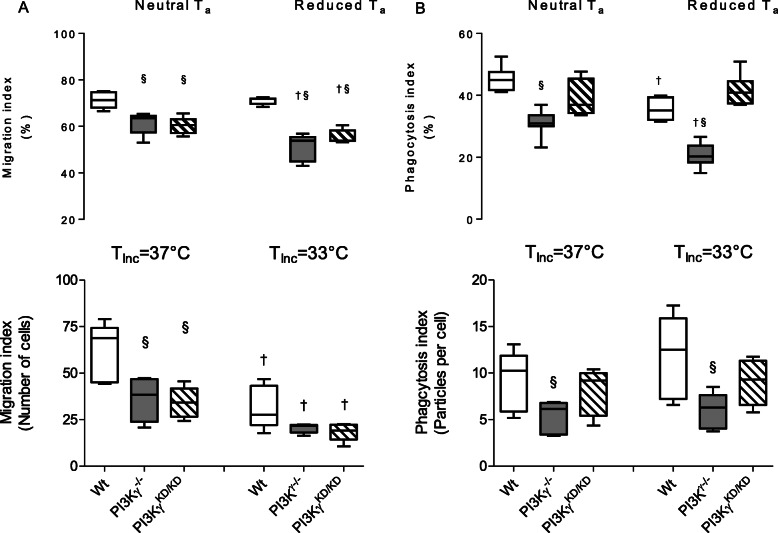
PI3Kγ-dependent suppression of microglial motility and phagocytic activity: **a** Lipid kinase-dependent reduction of migratory activity induced by brain injury (upper panel) and chemoattractant-stimulated migration of primary microglial cells (lower panel). Reduced ambient temperature as well as reduced temperature of incubation (*T*_Inc_ = 33 °C) led to a decline of microglial motility. **b** Lipid kinase-independent reduction of phagocytosis was not influenced by ambient/incubation temperatures: reduced number of Iba-1 positive cell with phagocytized zymosan particles in brains obtained from PI3Kγ^−/−^ mice (upper panel) as well as reduced uptake in number of zymosan particles by primary microglial cells obtained from PI3Kγ^−/−^ mice (upper panel). Values are presented as boxplots illustrating medians within boxes from first quartile to the third quartile and whiskers ranging from the 10th to the 90th percentiles (**a** and **b**: *n* = 5 for each group and experimental state. ^§ †^ *p* < 0.05, section sign indicates significant differences versus cells derived from wild-type mice kept under same T_a_ or T_Inc_, dagger sign indicates significant differences versus cells derived from mice kept under normal T_a_ or T_Inc_ at the same experimental state and genotype, two-way ANOVA, followed by Holm–Sidak test for post hoc multiple comparisons, each)

First, the in vitro cell motility was quantified toward migration to C5a added to bottom well of the transwell assay together with LPS stimulation. C5a acts as inflammatory peptide resulting in stimulation of microglial migration toward this chemo attractant. As demonstrated in Fig. 7a, PI3Kγ− deficiency as well as targeted knockout of the lipid kinase activity of PI3Kγ caused a markedly reduced migratory capacity by about 50% compared with cells derived from wild-type mice. A moderately reduced T_Inc_ provoked a further reduction in directed motility of primary microglial cells, whereas the PI3Kγ-related migratory alteration remained preserved.

To assess the effect of PI3Kγ^−^ deficiency on directed cell motility in vivo, a wound healing assay using focal stab-injury [40] was carried out. Whereas the baseline number of microglial cells was similar in wild type, PI3Kγ deficient, and PI3Kγ-kinase dead brains (Table 4 Suppl.), migration of microglia in direction of the focal stab injury was clearly reduced in the brains from PI3Kγ mutants as indicated by reduced microglial cell numbers in the inner region of interest (see Fig. 7a, Suppl. Fig. 4) of the injury site, which was markedly reduced in mice kept at reduced T_a_. Taken together, the present data indicate an inhibitory role of reduced T_a_ for the directional migration/chemotaxis of microglial cells from PI3Kγ mutants.

Next, the role of modified T_a_ ambient temperature for phagocytosis, another essential function of microglia, was analyzed. First, efficiency of phagocytosis was quantified by in vitro incubation of microglia with FITC-labeled Zymosan particles and subsequent counting of incorporated particles inside the microglial cells. PI3Kγ deficiency caused a distinct decrease of phagocytosis of microglial cells under normal T_Inc_ (Fig. 7b, Suppl. Fig. 5). Under reduced T_Inc_, quite similar effects have been ascertained. In vivo analysis of phagocytosis was performed by intracerebral administration of Zymosan particles. Under neutral T_a_, counting the number of cells with phagocytosed particles revealed a reduction of microglial phagocytic activity in the brains derived from PI3Kγ^−/−^ mice. Reduced T_a_ caused an additional distinct inhibition of phagocytic activity which was even more pronounced in PI3γ-deficient mice.

## Discussion

Our study identifies ambient temperature as a major impact factor for extent and clinical course of LPS-induced SIRS and concomitant blood-brain barrier breakdown as a key event in the development of SAE. We studied LPS-induced SIRS at a T_a_ of 30 °C (within thermoneutral zone for mice [46]) and at the upper edge of the recommended standard housing temperatures for laboratory mice, e.g., at 26 °C [30, 47]. Importantly, recent guidelines for preclinical studies in sepsis research [48] and cerebrovascular research [49–51], with the emphasis to improve reproducibility and translational impact, neglected the impact of ambient temperature on pathogenesis of inflammatory diseases or did not consider that their recommended lower baseline limit for housing temperatures of small rodents causes chronic “cold” stress [52, 53]. Furthermore, ignoring the role of ambient temperature on basal physiological responsiveness in small animals frequently used in preclinical sepsis models leads to far-reaching repercussions of profound misinterpretation. Indeed, usage time course of body temperature for prognosis prediction under chronic cold stress for mice restricts statements of numerous pertinent reports [54–58] merely on the subgroup of septic patients endangered for cold challenge leading to accidental hypothermia [59]. The recent findings in mice showed that a reduced ambient temperature exacerbates SIRS-induced cardiac autonomic dysregulation and myocardial dysfunction [60]. This study in the heart supports the present results and conclusions on the brain response to cold stress.

We show for the first time that a reduction of T_a_ of only 3–4° below the lower critical T_a_ for mice [61] increases the severity of BBB injury as a consequence of LPS-induced SIRS. This was clearly associated with a temporary disturbance of thermoregulation as a fundamental homeostatic function of all mammals because body core temperature was markedly reduced early after LPS-induced SIRS, and hypothermia persisted throughout the observed period (Fig. 2). Occurrence of hypothermia as a result of sepsis and overwhelming systemic inflammation characterizes a specific state of disturbed thermoregulation. Indeed, experimental and clinical studies clearly showed that severity of inflammation determines the magnitude of displacement from normothermia, e.g., mild to moderate SIRS induced by low and medium dosages of LPS provokes fever, whereas severe/life-threatening SIRS induces hypothermia [62–64] as a result of decreased threshold of body temperature for activation of metabolic heat production [62, 65]. The mechanisms regulating hypothermia are not fully understood, but cytokines such as TNF-α, interleukins (ILs), and interferon-gamma have been shown to induce or modulate hypothermia [66]. The herein presented data suggest that TNF-α may contribute to the hypothermic response because of a similar temporal profile. LPS-induced microglial activation may directly alter thermoregulation during systemic inflammation possibly by inducing cerebral endothelial activation [67–69]. There is ample evidence that fever can be triggered by peripheral or intracerebrally administered (low-dose) LPS [70–72]. In contrast, hypothermic response to inflammation can be triggered only by (high-dose) LPS administered extra-cerebrally, i.e., systemically [73, 74]. Apparently, other factors play a relevant role in the extent of hypothermia early after LPS-induced SIRS. Clearly, T_a_ determines depth of T_c_ reduction (Fig. 2). Therefore, hypothermic response appears to be a consequence of maladaptive thermoregulation leading to hypometabolism in order to avoid hypoxia [75].

PI3Kγ-dependent differences in extent of sickness suggest that disturbance in behavioral thermoregulation may contribute to the manifestation of hypothermia. Small rodents such as mice need an increased metabolic rate and periodic motor activity for appropriate heat production to maintain homeothermy because of its unfavorable surface area versus mass ratio [61, 76]. Mice develop hypothermia when locomotor activity is diminished due to consequences of sickness induced by infection [77]. Our findings show that mice kept at reduced T_a_ developed an exacerbated and prolonged hypothermia although they exhibited a markedly enhanced sympathetic tone. This might be related to a stronger impairment of thermoregulation with torpor-like traits elicited by LPS-induced SIRS [78], in addition to LPS-induced inhibition of brown adipose tissue thermogenesis [79].

In a previous study, we have shown that the kinase-independent control of cAMP phosphodiesterase activity by PI3Kγ acts as a crucial mediator of microglial cell activation, MMP expression, and subsequent BBB deterioration [5]. The data obtained in the current study suggest that an aggravated BBB breakdown observed in mice kept at reduced T_a_ during LPS-induced SIRS results from an intensified LPS-induced proinflammatory microglial response. This response is accompanied by the pronounced upregulation of brain MMP expression and perivascular MMP-9 release leading to increased PMN invasion with altered microglial migration and phagocytosis. These processes appear to be widespread because we made similar findings in quite different brain regions. Enhanced plasma protein extravasation in brains obtained from PI3Kγ-deficient mice kept under reduced T_a_ suggests that the genotype-related differences in BBB breakdown appear to be related to microglial activation in response to systemic inflammation and concomitant brain tissue MMP upregulation. Compelling evidence suggests that early after SIRS manifestation, constitutive proteases are activated and begin the process of disassembling the extracellular matrix and opening the BBB [80, 81]. Immunohistological evaluation revealed that there is an increased number of Iba-1 positive cells which co-express MMP-9 in brains obtained from PI3Kγ-deficient mice kept under reduced T_a_ (Fig. 6). Our previous results revealed that the enhanced MMP-9 activity is of microglial origin and provoked by a deficient suppression of cAMP-dependent proinflammatory signaling in PI3Kγ-deficient mice [5]. MMP-9 is known to act as an executing protease for degrading matrix substrates and interrupting cell–cell or cell–matrix homeostatic interactions, which may directly trigger anoikis-like neuronal cell death by interrupting cell–matrix survival signaling [82]. The current findings of aggravated SIRS-induced BBB impairment associated with reduced T_a_ are clearly PI3Kγ-dependent and induce an enhanced invasion of blood-born immune cells and an increased rate of apoptosis, especially in the hippocampus, when the suppressive effect of PI3Kγ on cAMP as a critical mediator of immune cell functions is absent [5, 32, 83].

BBB impairment was revealed by EB extravasation into brain tissue indicative for plasma protein passage into brain tissue. EB remains the most commonly used marker of BBB integrity [84, 85]. Among the strengths of EB for studying BBB integrity are its immediate visibility and quantifiability allowing for group comparisons without usage of radioactive indicators [5, 36, 86]. However, there is now compelling evidence that results estimated by EB technique need to be viewed critically. Specifically, the general understanding is that EB binds tightly and nearly exclusively to plasma albumin. Therefore, EB extravasation into brain tissue is viewed as indication of an increased BBB permeability [87]. However, there is evidence that EB is additionally bound on some other plasma proteins, albeit with reduced physical binding [88, 89]. Furthermore, with the conventionally used solution of 2% EB (4 ml/kg) for in vivo administration, the plasma concentration of EB would not have exceeded the maximum binding capacity of albumin [84]. This is the technique we used in the present study. Nevertheless, because of the mode of binding, it has to be considered that a small fraction of free dye (0.11–0.31%) remained present and may be responsible for a certain level of background [85, 89, 90]. Other aspects of in vivo EB administration like toxicity or insufficient protein binding because of shortened contact time appear to be irrelevant for the results presented herein due to the experimental design chosen: Reported toxic effects were registered days or weeks after EB administration (overview is given in [84]); EB binding equilibration after bolus injection was reached within 5 min [91]. These temporal profiles do not apply in the present experimental conditions.

Causal relations responsible for associated exacerbated brain injury cannot be drawn conclusively. Indeed, this study is limited in detailed mechanistic explanation of microglial role in BBB alterations. Future work needs to include approaches for fine-grained analysis of microglia spatial and temporal heterogeneity on the single-cell level [6]. Nevertheless, a reduced ability of directed motility and diminished phagocytic activity in the brains obtained from PI3Kγ-deficient mice kept under reduced T_a_ suggest that these altered cell functions contribute to the phenotype of enhanced structural and functional cerebral disturbance leading to aggravated SAE symptoms. We identified the lipid kinase activity of PI3Kγ as an essential mediator of microglial migration [92]. Furthermore, diminished microglial phagocytic activity appears to contribute to the enhanced proinflammatory brain response to LPS-induced SIRS in PI3Kγ-deficient mice kept under reduced T_a_ because microglial phagocytosis represents a key factor for limiting excessive proinflammatory activation by clearance of dying cells and debris in injured brain tissue [14, 93, 94].

The agreement between our findings obtained from in vivo experiments performed in mice and cell culture assays regarding cell motility and phagocytosis activity supports our inference on the impact of the interactions of microglial cells, the genotype, and environmental temperature on systemic inflammation and severity of brain injury. It has been shown that many features of microglial activation can be reproduced in primary cell culture [95]. Nevertheless, neonatal microglial isolation for cell culturing is a challenge because several necessities alter the basic environmental conditions with sustained functional consequences: (i) separation of microglia from neighboring cells and the tissue matrix requires extensive tissue damage resembling microglial features typical for injured tissue [96, 97]; (ii) high purity of microglial cells in cell culture (> 95%) is one of the intended objectives for mechanistic approaches and one of the most popular strengths. However, this also abolishes the interaction with surrounding neurons, other glia cells, and extracellular matrix, with far-reaching consequences on microglial response patterns [14, 98]; (iii) in vitro survival and increase of yielded microglia necessitate serum supplement, which consequently induces microglial activation [37, 99].

Therefore, interpretation of results obtained from microglial cell culture experiments requires caution. Comparative discussion, as we did here, with corresponding experimental approaches in similar animal models allows critical assessment of the experimental data and reduces the risk of misinterpretation.

In addition, it is necessary to take into account the typically lower cell yield during neonatal microglial isolation. This creates challenges in getting sufficient cell numbers to perform primary microglial cell culture experiments. Therefore, (i) a rather high number of animals must be sacrificed for each experiment. Keeping in mind the necessity for enforced application of high animal welfare standards, an ethical evaluation and consideration of the scientific significance, e.g., a scientific validation for harm-benefit analysis for each and every experiment has to be considered [100, 101]; (ii) in order to warrant a sufficient cell number and appropriate purity of neonatal microglia for in vitro approaches, different methods have been described and comprehensively reviewed [99, 102, 103]. The herein used method is based on the most frequently used differential adherence method. Mixed glial cultures are obtained and allowed to grow to confluency. The microglial cells are then separated from the adherent astrocyte layer by gently shaking [104]. The resulting microglial cell population appears highly pure and exhibits a largely uniform phenotype compared with the diversity of phenotypes observed in vivo [105]. Therefore, such in vitro approaches have allowed for an intimate exploration of key activation and signaling pathways [106].

Some additional methodological implications have to be considered. Categorization and classification of microglia and phagocytic indexing by Iba1 staining appear to be appropriate methodological approaches [107] used in numerous studies (see below). Specifically, Iba1 is known to be constitutively expressed by microglia within the brain parenchyma. Iba1 is only moderately expressed by quiescent ramified microglia and not at all by astrocytes, oligodendrocytes, or neurons [108]. Iba1 is a protein that acts to modulate membrane-ruffling changes during microglial activation [108]. Therefore, Iba1 appears to be a suitable indicator for microglial indexing and has been extensively used to identify, count, or gage activation of microglia within the CNS [109–113]. Furthermore, while the morphometric analysis of microglia for indexing the microglial activation status was the first approach characterizing different microglial response states [114–116], it remains also an appropriate measure for microglial activation, in particular if the expression level of several molecular biomarkers, such as Iba1, is mostly increasing with microglial activation [14]. In addition, brain histological analysis revealed a clear regional pattern, which enhanced the significance of our findings on the impact of ambient temperature on inflammation-induced encephalopathy in endotoxemic mice. In addition, we used methods for analyses of two key microglial cell functions, i.e., migration and phagocytosis, which are both well-documented [41, 117–121] and well-established in our lab, having been used successfully for years [40, 92, 122].

We consider the following aspects as strengths of the experimental design used herein: (i) two experimental groups of mice kept at neutral (30 ± 0.5 °C) or moderately lowered (26 ± 0.5 °C) T_a_. (ii) Three different genotypes were used which together are suited to clarify mechanistically the role of the signaling protein PI3Kγ [5, 8, 92, 94, 122–124]. Therefore, the two different PI3Kγ’s main functions were studied: PI3Kγ lipid kinase activity producing of phosphatidylinositol 3,4,5-trisphosphate and protein kinase Akt/PKB activation, and/or kinase-independent control of cAMP phosphodiesterase activity. (iii) Using the in vitro approach with pure microglia cell cultures derived from the three mice genotypes allowed to clarify the role of microglia and PI3Kγ more mechanistically.

Taken together, we put forward that the multifaceted methodological approach and the experimental design deployed in this study conclusively validate our main hypothesis that thermoregulatory response to hypothermia (reduced T_a_) aggravates SAE in PI3Kγ-dependent manner.

## Conclusions

Our findings underline the importance of ambient temperature as a frequently neglected yet crucial environmental condition in translational inflammatory/infectious diseases research. The major significance of our findings is that a modest variation of an easily controllable parameter, i.e., the ambient temperature, led to a serious impact on the course of SAE. Furthermore, our data reveal the signaling protein PI3Kγ as a critical mediator of key microglial cell functions involved in LPS-induced BBB injury and accompanying neuroinflammation. PI3Kγ serves a protective role in that it suppresses MMP release, maintains microglial motility, and reinforces phagocytosis leading to improved brain tissue integrity.

Thus, this study substantiates the importance of controlling T_a_ tightly to prevent serious bias in results from preclinical animal research on inflammation/infection. Accounting for T_a_ will improve the predictive power and value of the neuroinflammatory research and help overcome the “replication crisis” [125].

## Supplementary information

**Additional file 1.**

## Data Availability

The datasets used and/or analyzed during the current study are available from the corresponding author upon reasonable request.

## Abbreviations

5′-AMP: Adenosine monophosphate
Akt: Protein kinase B
BBB: Blood–brain barrier
C5a: Complement component 5a
cAMP: Cyclic adenosine monophosphate
cDNA: Complementary DNA
CNS: Central nervous system
DAPI: 4′,6-diamidino-2-phenylindole
DMEM: Dulbecco/Vogt modified Eagle’s minimal essential medium
EB: Evans blue
ECG: Electrocardiography
EDTA: Ethylenediaminetetraacetic acid
FCS: Fetal calf serum
FITC: Fluoresceinisothiocyanate
GAPDH: Glyceraldehyde 3-phosphate dehydrogenase
HR: Instantaneous heart rate
IL-6: Interleukin 6
IL-10: Interleukin 10
LPS: Lipopolysaccharides
MCP-1: Monocyte chemoattractant protein 1
MMPs: Matrix metalloproteinases
mRNA: Messenger RNA
PBS: Phosphate-buffered saline
PFA: Paraformaldehyde
PI3Kγ: Phosphoinositide 3-kinase γ
PI3Kγ^−/−^: PI3Kγ knockout
PI3Kγ^KD/KD^: Mutation in the PI3Kγ gene causing loss of lipid kinase activity
PKA: Protein kinase A
PMN: Polymorphonuclear neutrophil
qPCR: Quantitative PCR
SAE: Sepsis-associated encephalopathy
SIRS: Systemic inflammatory response syndrome
Ta: Ambient temperature
TC: Core body temperature
TInc: Incubation temperature
TNF-α: Tumor necrosis factor alpha
