# Supplementary material for: Impact of ambient temperature on inflammation-induced encephalopathy in endotoxemic mice—role of phosphoinositide 3-kinase gamma
Source: J Neuroinflammation. 2020 Oct 7;17:292. doi: 10.1186/s12974-020-01954-7 (PMC7541275; doi:10.1186/s12974-020-01954-7)
Supplement: Supplementary file 1 — Additional file 1. [file 12974_2020_1954_MOESM1_ESM.docx]

**Supplementary material:**

Guang-Ping Lang: Reduced ambient temperature enhances inflammation-induced encephalopathy

*SUPPLEMENTAL MATERIAL*

**Results**

Table 1 Suppl: Clinical severity score (according to [[1](#_ENREF_1)]).

|  |  |  |
| --- | --- | --- |
|  | ***Baseline*** | ***24h-LPS*** |
| *Neutral T_a_* |  |  |
| Wt | 1.0 (1.0; 1.0) | 1.5 (1.0; 2.0)* |
| PI3Kγ^-/-^ | 1.0 (1.0; 1.0) | 2.0 (1.5; 2.0)* |
| PI3Kγ^-KD/KD^ | 1.0 (1.0; 1.0) | 1.0 (1.0; 2.0)* |
|  |  |  |
| *Reduced T_a_* |  |  |
| Wt | 1.0 (1.0; 1.0) | 2.0 (1.5; 2.0)* |
| PI3Kγ^-/-^ | 1.0 (1.0; 1.0) | 2.5 (2.0; 3.0)*^†§^ |
| PI3Kγ^-KD/KD^ | 1.0 (1.0; 1.0) | 2.0 (2.0; 2.0)* ^†^ |

(Values are given as medians as well as the first quartile and third quartile in parentheses, n=4-6 animals per each group and experimental state. * ^§^ †p < 0.05, * significant difference between baseline and LPS-stimulated state within each group, ^§^ significant difference versus wild type (Wt) mice kept under same ambient temperature, †significant differences versus mice kept under neutral ambient temperature).

Table 2 Suppl: Quantitative morphometric analysis of cell density of activated microglia (assessed by cell shape characteristics [[2](#_ENREF_2), [3](#_ENREF_3)]) in different brain structures

| **Groups** | **Cortex** | | **Hippocampus** | | **Thalamus** | |
| --- | --- | --- | --- | --- | --- | --- |
|  | ***Baseline*** | ***24h-LPS*** | ***Baseline*** | ***24h-LPS*** | ***Baseline*** | ***24h-LPS*** |
| *Neutral T_a_* |  |  |  |  |  |  |
| Wt | 0.04 (0.03, 0.04) | 0.28 (0.2, 0.36) * | 0.06 (0.05, 0.07) | 0.27 (0.22, 0.33) * | 0.09 (0.05, 0.09) | 0.3 (0.28, 0.31) * |
| PI3Kγ^-/-^ | 0.05 (0.02, 0.08) | 0.4 (0.28, 0.51) * | 0.05 (0.03, 0.09) | 0.5 (0.4, 0.56) *^§^ | 0.15 (0.12, 0.18) | 0.6 (0.58, 0.65) *^§^ |
| PI3Kγ^-KD/KD^ | 0.04 (0.03, 0.06) | 0.32 (0.3, 0.35) * | 0.06 (0.03, 0.12) | 0.42 (0.39, 0.44) * | 0.12 (0.09, 0.16) | 0.41 (0.36, 0.47) * |
|  |  |  |  |  |  |  |
| *Reduced T_a_* |  |  |  |  |  |  |
| Wt | 0.03 (0.02, 0.09) | 0.39 (0.3, 0.43) * | 0.04 (0.02, 0.09) | 0.38 (0.33, 0.38) * | 0.13 (0.05, 0.19) | 0.46 (0.44, 0.48) *† |
| PI3Kγ^-/-^ | 0.05 (0.04, 0.06) | 0.51 (0.38, 0.38) * | 0.06 (0.05, 0.08) | 0.55 (0.5, 0.5) *^§^ | 0.09 (0.08, 0.11) | 0.61 (0.53, 0.63) * |
| PI3Kγ^-KD/KD^ | 0.06 (0.04, 0.08) | 0.33 (0.28, 0.34) * | 0.07 (0.06, 0.09) | 0.35 (0.28, 0.42) * | 0.1 (0.07, 0.1) | 0.43 (0.39, 0.43) * |

(Cell counting for assessment of microglial cell activation: Cells were classified as ramified, amoeboid, unipolar and bipolar. Ramified (normal) microglial cells are defined by thin, slender, radially projecting processes with well-developed ramifications. Amoeboid microglial cells are defined as having large soma, and short, thick and radially projecting processes. Unipolar and bipolar microglial cells were defined as having one or two thick processes with well-developed ramifications. Values are given as medians as well as the first quartile and third quartile in parentheses, n=4-6 animals per each group and experimental state. * ^§^ †p < 0.05, * significant difference between baseline and LPS-stimulated state within each group, ^§^ significant difference versus wild type (Wt) mice kept under same ambient temperature, †significant differences versus mice kept under neutral ambient temperature).

Table 3 Suppl: Regional distribution of MMP-9 positive cells, TUNEL positive cells and invading polymorphonuclear leukocytes (PMN).

|  | **Cortex** | | **Hippocampus** | | **Thalamus** | |  |
| --- | --- | --- | --- | --- | --- | --- | --- |
|  | ***Baseline*** | ***24h-LPS*** | ***Baseline*** | ***24h-LPS*** | ***Baseline*** | ***24h-LPS*** |  |
| MMP-9 positive cells | |  |  |  |  |  |  |
| *Neutral T_a_* |  |  |  |  |  |  |  |
| Wt | 0.05 (0.05, 0.06) | 0.34 (0.34, 0.37) * | 0.14 (0.11, 0.14) | 0.79 (0.69, 0.94) * | 0.05 (0.04, 0.07) | 0.42 (0.33, 0.49) * |  |
| PI3Kγ^-/-^ | 0.07 (0.04, 0.1) | 0.46 (0.44, 0.5) * | 0.22 (0.11, 0.36) | 0.72 (0.65, 1.01) * | 0.08 (0.05, 0.11) | 0.53 (0.37, 0.58) * |  |
| PI3Kγ^-KD/KD^ | 0.07 (0.05, 0.15) | 0.51 (0.45, 0.56) * | 0.14 (0.11, 0.22) | 1.44 (1.08, 1.8) * | 0.05 (0, 0.11) | 0.53 (0.37, 0.58) * |  |
| *Reduced T_a_* |  |  |  |  |  |  |  |
| Wt | 0.07 (0.05, 0.11) | 0.46 (0.42, 0.5) * | 0.07 (0, 0.14) | 0.72 (0.65, 0.79) * | 0.03 (0, 0.07) | 0.53 (0.53, 0.53) * |  |
| PI3Kγ^-/-^ | 0.1 (0.1, 0.11) | 0.73 (0.71, 0.75) *^†§^ | 0.22 (0.11, 0.29) | 1.23 (0.76, 0.97) *^†§^ | 0.05 (0.04, 0.05) | 0.32 (0.26, 0.28) *^†§^ |  |
| PI3Kγ^-KD/KD^ | 0.1 (0.07, 0.11) | 0.61 (0.57, 0.66) *^§^ | 0.14 (0.11, 0.18) | 0.87 (0.58, 1.3) *^†§^ | 0.08 (0.05, 0.12) | 0.37 (0.3, 0.45) *^§^ |  |
|  | |  |  |  |  |  |  |
| TUNEL positive cells | |  |  |  |  |  |  |
| *Neutral T_a_* |  |  |  |  |  |  |  |
| Wt | 0.27 (0.24, 0.29) | 0.49 (0.44, 0.55)* | 0.58 (0.54, 0.58)^$^ | 2.24 (2.13, 2.31)*^$^ | 0.05 (0.04, 0.07) | 0.42 (0.33, 0.49) * |  |
| PI3Kγ^-/-^ | 0.32 (0.28, 0.34) | 0.64 (0.56, 0.68)* | 1.01 (0.9, 1.08)^$^ | 1.37 (1.3, 1.59)*^§$^ | 0.08 (0.05, 0.11) | 0.53 (0.37, 0.58) * |  |
| PI3Kγ^-KD/KD^ | 0.29 (0.18, 0.42) | 0.46 (0.4, 0.5) | 0.5 (0.36, 0.61)^$^ | 1.01 (0.87, 1.19)*^§$^ | 0.05 (0, 0.11) | 0.53 (0.37, 0.58) * |  |
| *Reduced T_a_* |  |  |  |  |  |  |  |
| Wt | 0.29 (0.27, 0.33) | 0.68 (0.66, 0.71)* | 0.72 (0.43, 1.05)^$^ | 1.59 (1.55, 1.62)*^†$^ | 0.13 (0.11, 0.16) | 0.29 (0.26, 0.32)* |  |
| PI3Kγ^-/-^ | 0.29 (0.28, 0.33) | 1 (0.93, 1.1)*†§ | 1.3 (1.23, 1.33) | 2.81 (2.74, 3.03)*^†§$^ | 0.24 (0.2, 0.29) | 0.66 (0.55, 0.68)*^§^ |  |
| PI3Kγ^-KD/KD^ | 0.29 (0.23, 0.34) | 0.46 (0.4, 0.5)*§ | 0.65 (0.58, 0.76)^$^ | 1.95 (1.84, 2.09)*^$^ | 0.21 (0.18, 0.22) | 0.42 (0.39, 0.42)* |  |
|  |  |  |  |  |  |  |  |
| Polymorphonuclear leukocytes | |  |  |  |  |  |  |
| *Neutral T_a_* |  |  |  |  |  |  |  |
| Wt | 0.2 (0.2, 0.22) | 0.83 (0.73, 0.86) * | 0 (0, 0.07) | 0.29 (0.29, 0.36) * | 0.16 (0.11, 0.18) | 0.58 (0.55, 0.6) * |  |
| PI3Kγ^-/-^ | 0.1 (0.1, 0.15) | 1.61 (1.34, 1.61) * | 0 (0, 0.07) | 1.15 (0.87, 1.23) * | 0.05 (0.05, 0.11) | 1 (0.97, 1.08) * |  |
| PI3Kγ^-KD/KD^ | 0.24 (0.17, 0.27) | 0.88 (0.78, 0.98) * | 0.14 (0.07, 0.22) | 1.59 (1.52, 1.8) * | 0 (0, 0.13) | 1.05 (0.89, 1.13) * |  |
| *Reduced T_a_* |  |  |  |  |  |  |  |
| Wt | 0.44 (0.23, 0.46) | 1.08 (0.95, 1.1) * | 0.29 (0.22, 0.51) | 1.44 (1.3, 1.52) * | 0.37 (0.37, 0.39) | 0.89 (0.87, 0.92) * |  |
| PI3Kγ^-/-^ | 0.29 (0.27, 0.32) | 2.64 (2.52, 2.71) *^†§^ | 0.14 (0.14, 0.22) | 2.74 (2.53, 3.1) *^†§^ | 0.21 (0.18, 0.21) | 1.68 (1.66, 1.92) *^†§^ |  |
| PI3Kγ^-KD/KD^ | 0.05 (0.05, 0.81) | 0.78 (0.76, 0.27) *^§^ | 0 (0, 0.79) | 0.72 (0.65, 0.22) *^†§^ | 0.05 (0.05, 0.97) | 0.95 (0.87, 0.13) *^§^ |  |
|  |  |  |  |  |  |  |  |
|  |  |  |  |  |  |  |  |

(Values are given as medians, first quartile and third quartile in parentheses. n=4 at each group and experimental state.* ^† § $^ p < 0.05, * significant differences versus baseline for the same genotype and experimental state, ^†^ significant differences versus mice kept under neutral T_a_ at the same experimental state, ^§^ significant differences versus wild type mice kept under same T_a_, ^$^ significant differences versus cortex & thalamus, two-way ANOVA, followed by Holm–Sidak test for post hoc multiple comparisons, each).

Table 4 Suppl: Number of microglial cells in brain cortex

|  |  |
| --- | --- |
|  | ***Baseline*** |
| *Neutral T_a_* |  |
| Wt | 4483 (4447; 4554) |
| PI3Kγ^-/-^ | 4132 (4099; 4371) |
| PI3Kγ^-KD/KD^ | 4536 (4362; 4653) |
|  |  |
| *Reduced T_a_* |  |
| Wt | 4464 (4259; 4582) |
| PI3Kγ^-/-^ | 4303 (4160; 4664) |
| PI3Kγ^-KD/KD^ | 4546 (4399; 4615) |

(Values are given as medians, first quartile and third quartile in parentheses.

n=7-8 for each group and experimental state. One-way ANOVA).


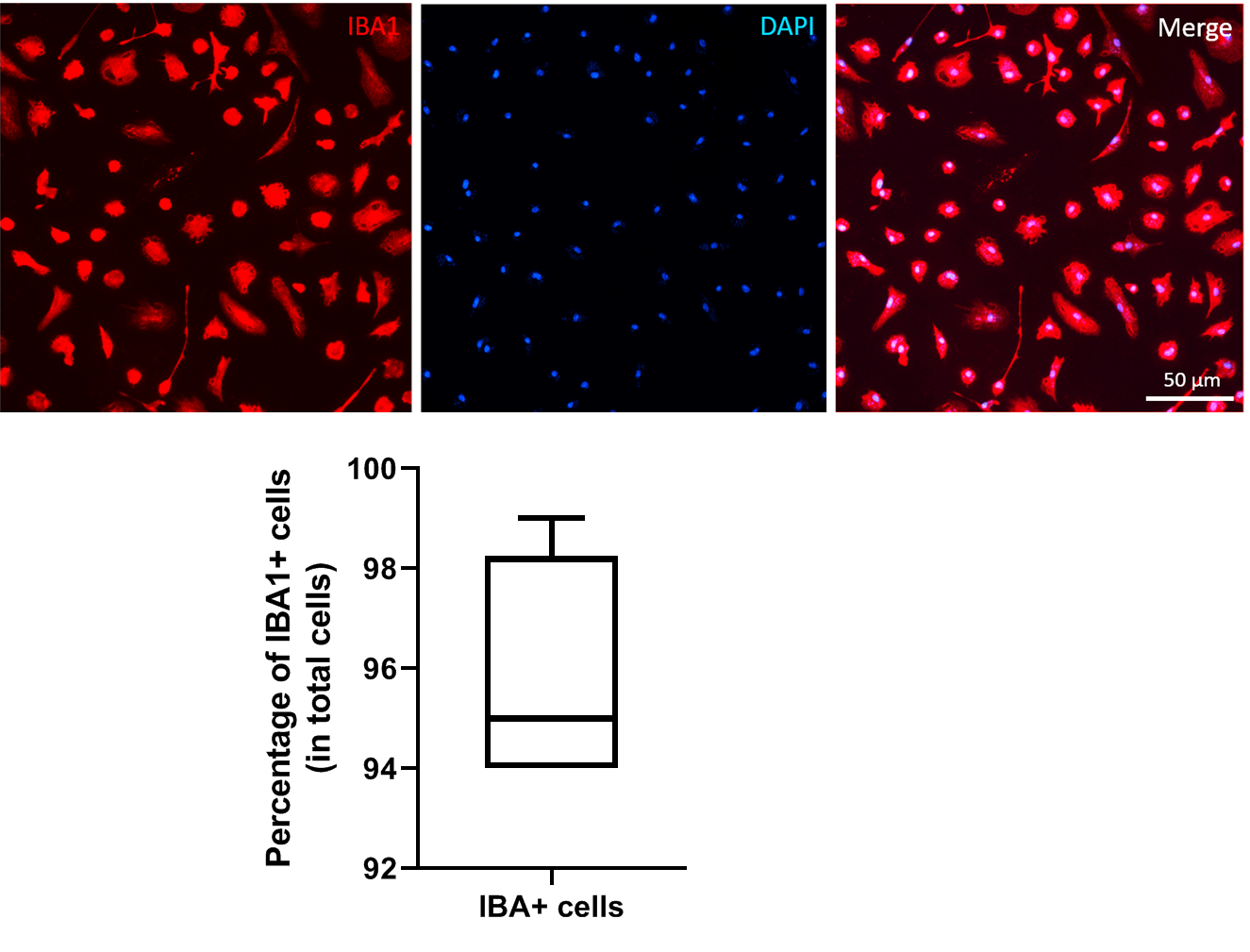


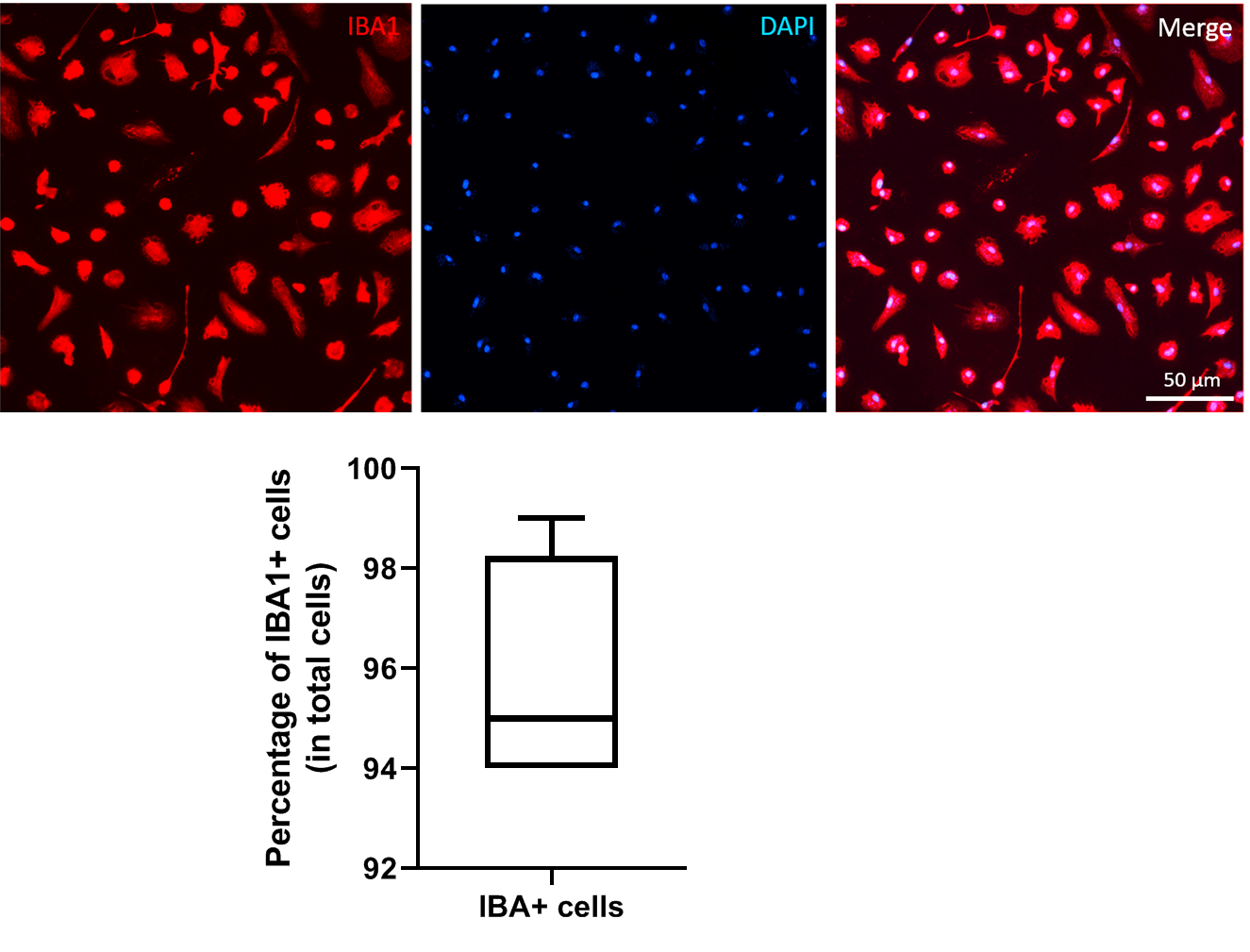


Suppl. Fig. 1: The purity of isolated primary microglial cells from newborn pups with each isolation method was determined by the percentage of IBA1-positive cells (red color) in total cells (indicated by immunocytochemical staining using DAPI, blue color). N=6, 5 randomly selected fields were used for quantification. Values are presented as boxplots illustrating medians within boxes from first quartile to the third quartile and whiskers ranging from the 10th to the 90th percentiles.


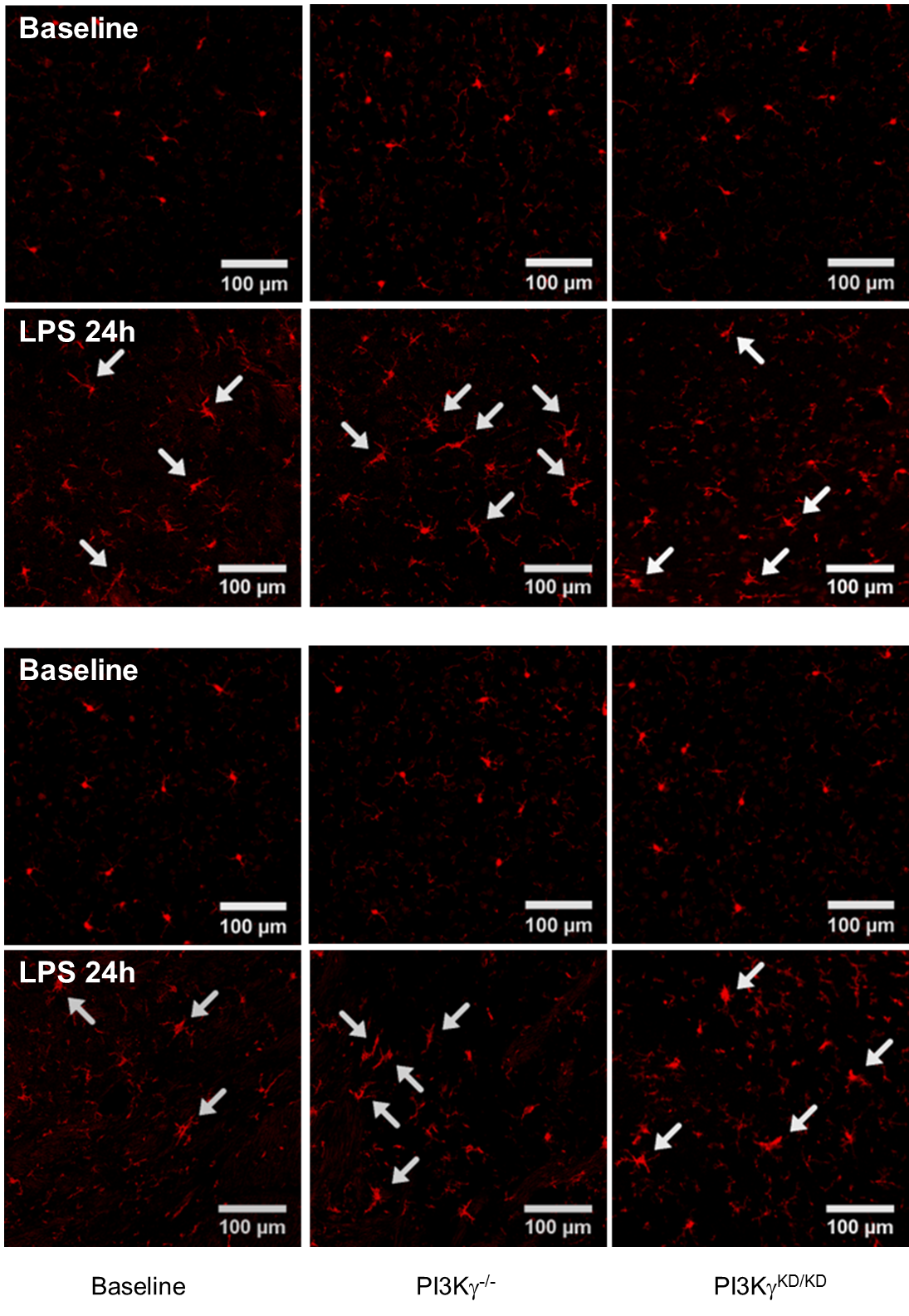


Suppl. Fig. 2: Marked increase of microglial cell activation due to LPS-induced SIRS in brains of mice kept under neutral T_a_ (lower panel) and reduced T_a_ (upper panel; representative photomicrographs). Note the increased number of Iba1-positive cells with altered, mainly polarized shape 24h after LPS administration.


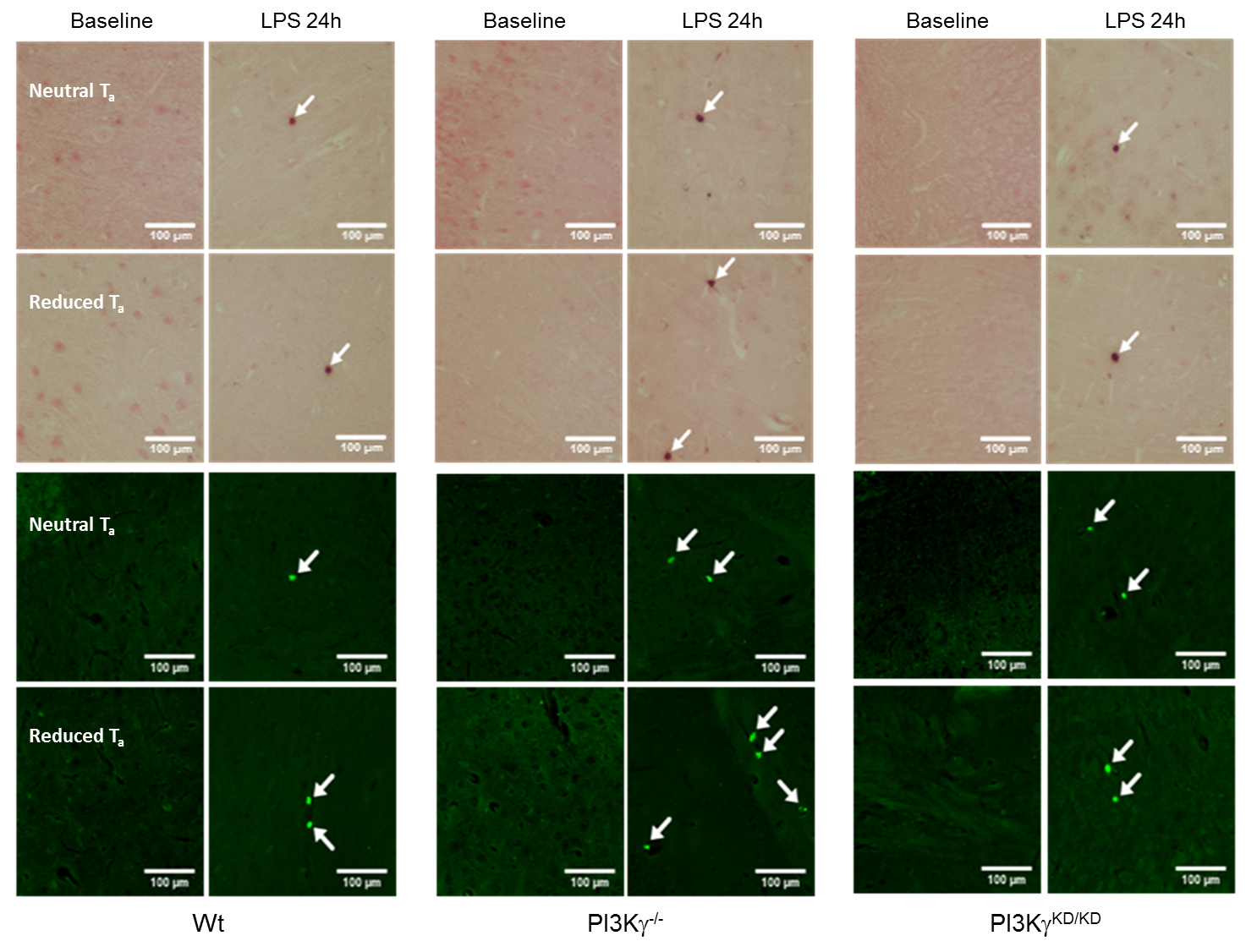


Suppl. Fig. 3: Representative photomicrographs showing an increased number of TUNEL positive cells (arrows) and of invading polymorphonuclear cells (arrows) appearing mainly in the brains obtained from PI3Kγ-deficient mice kept under reduced T_a_ (26°C) 24h after LPS administration.


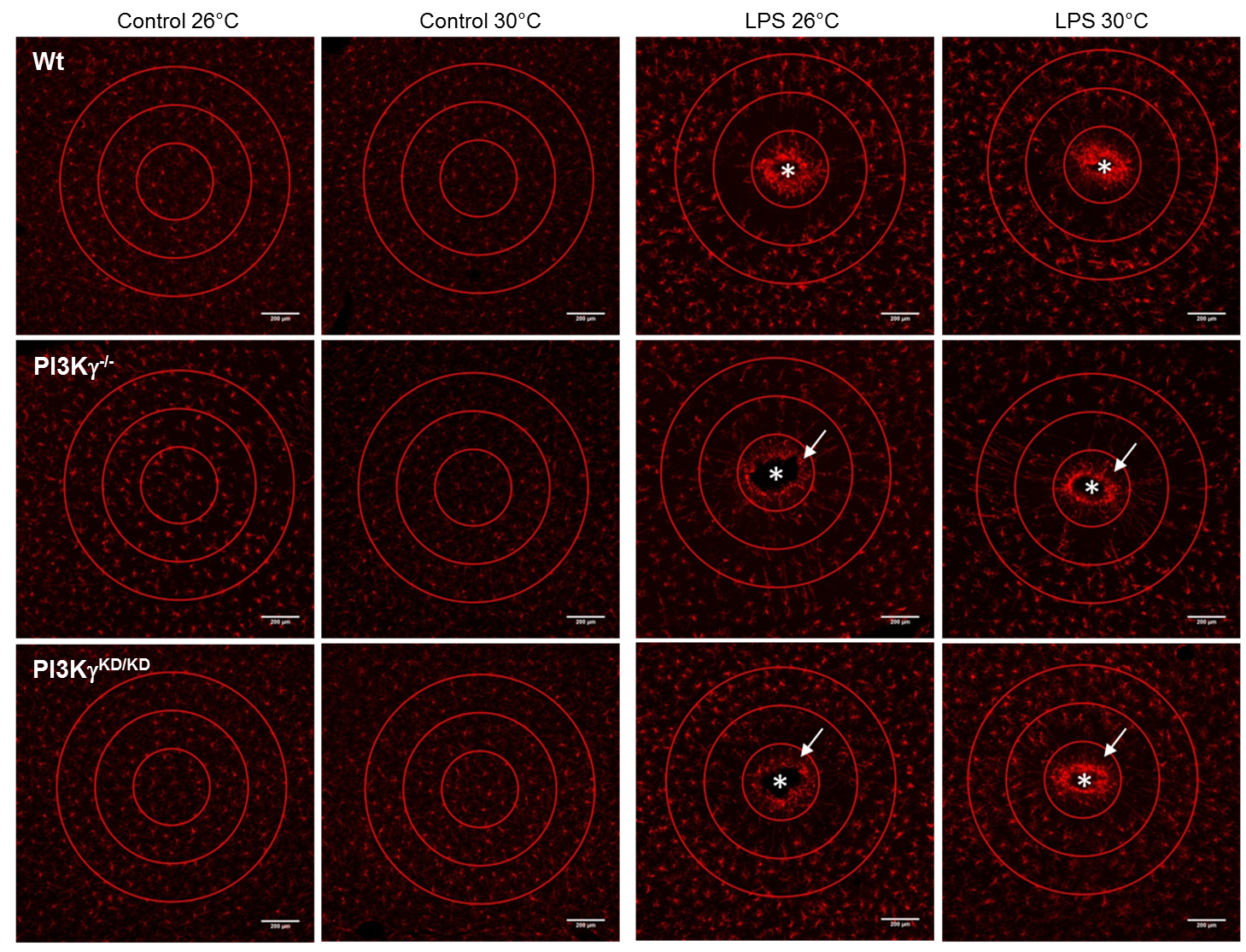


Suppl. Fig. 4: Depiction of microglial (Iba1 positive) cell migration toward induced brain tissue injury in wild type (Wt) and PI3Kγ-deficient (PI3Kγ^-/-^) mice, as well as mice carrying a targeted mutation in the PI3Kγ gene causing loss of lipid kinase activity (PI3Kγ^KD/KD^). Right panel: * indicate central cavity produced by focal stab injury. The three concentric circles mark the circumference of the three different voxels under consideration: Voxel 1, a cylinder with a diameter of 400 µm, center lying in the middle of injury, and an altitude of 40 µm; Voxel 2, hollow cylinder, subsequently on Voxel 1, with an inner diameter of 400 µm, an outer diameter of 800 µm, and an altitude of 40 µm; Voxel 3, hollow cylinder, subsequently on Voxel 2, with an inner diameter of 800 µm, an outer diameter of 1200 µm, and an altitude of 40 µm.). Left panel: representative pictures of naïve microglial cells. Note that the number of Iba1-positive cells is markedly reduced in the border region of the focal stab injury in brains obtained from PI3Kγ^-/-^ and PI3Kγ^KD/KD^ mice (arrows) compared with respective Wt brains (bar 200 μm).


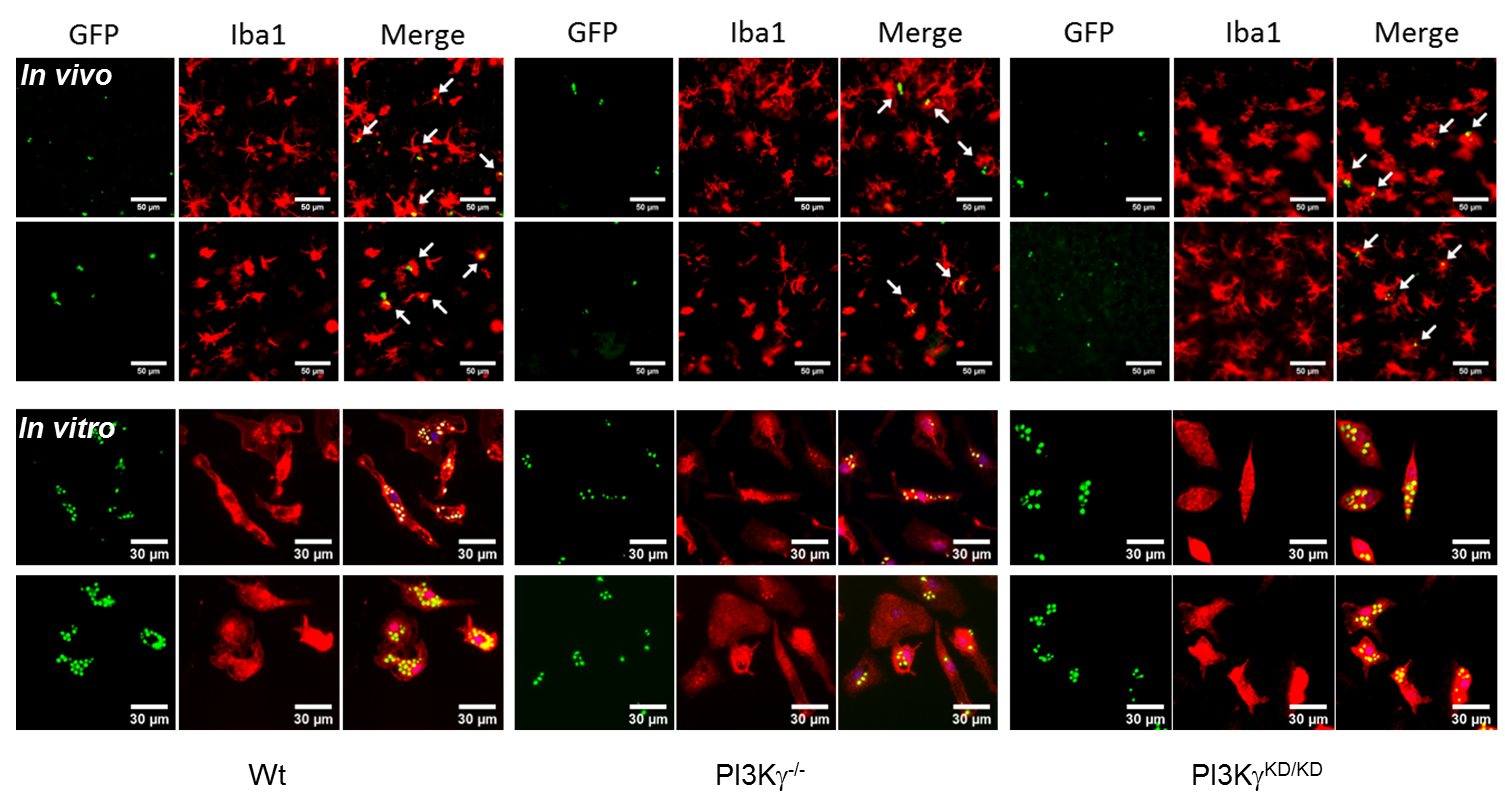


Suppl. Fig. 5: Depiction of microglial (Iba1 positive) cells with phagocytized Zymosan particles (GFP). Upper panel, in vivo study: brains obtained from wild type (Wt) and PI3Kγ-deficient (PI3Kγ^-/-^) mice as well as mice carrying a targeted mutation in the PI3Kγ gene causing loss of lipid kinase activity (PI3Kγ^KD/KD^). Lower panel, in vitro study: primary microglial cells obtained from Wt, PI3Kγ^-/^ and PI3Kγ^KD/KD^ mice. Note a reduced number of Iba-1 positive cell with phagocytized Zymosan particles in brains obtained from PI3Kγ^-/-^ mice as well as reduced uptake in number of Zymosan particles by primary microglial cells obtained from PI3Kγ^-/-^ mice (indicated by arrows) (upper panel, bar 50 μm; lower panel, bar 30 μm).

References

1. Gonnert FA, Recknagel P, Seidel M, Jbeily N, Dahlke K, Bockmeyer CL, Winning J, Losche W, Claus RA, Bauer M: **Characteristics of clinical sepsis reflected in a reliable and reproducible rodent sepsis model.** *J Surg Res* 2011, **170:**e123-134.

2. Kettenmann H, Hanisch UK, Noda M, Verkhratsky A: **Physiology of microglia.** *Physiol Rev* 2011, **91:**461-553.

3. Zhang F, Vadakkan KI, Kim SS, Wu LJ, Shang Y, Zhuo M: **Selective activation of microglia in spinal cord but not higher cortical regions following nerve injury in adult mouse.** *Mol Pain* 2008, **4:**15.
